# Advancing Land-Sea Conservation Planning: Integrating Modelling of Catchments, Land-Use Change, and River Plumes to Prioritise Catchment Management and Protection

**DOI:** 10.1371/journal.pone.0145574

**Published:** 2015-12-29

**Authors:** Jorge G. Álvarez-Romero, Robert L. Pressey, Natalie C. Ban, Jon Brodie

**Affiliations:** 1 Australian Research Council Centre of Excellence for Coral Reef Studies, James Cook University, Townsville, Queensland, Australia; 2 School of Environmental Studies, University of Victoria, PO Box 1700 STN CSC, Victoria British Columbia, Canada; 3 Centre for Tropical Water and Aquatic Ecosystem Research (TropWater), Catchment to Reef Research Group, James Cook University, Townsville, Queensland, Australia; University of Sydney, AUSTRALIA

## Abstract

Human-induced changes to river loads of nutrients and sediments pose a significant threat to marine ecosystems. Ongoing land-use change can further increase these loads, and amplify the impacts of land-based threats on vulnerable marine ecosystems. Consequently, there is a need to assess these threats and prioritise actions to mitigate their impacts. A key question regarding prioritisation is whether actions in catchments to maintain coastal-marine water quality can be spatially congruent with actions for other management objectives, such as conserving terrestrial biodiversity. In selected catchments draining into the Gulf of California, Mexico, we employed Land Change Modeller to assess the vulnerability of areas with native vegetation to conversion into crops, pasture, and urban areas. We then used SedNet, a catchment modelling tool, to map the sources and estimate pollutant loads delivered to the Gulf by these catchments. Following these analyses, we used modelled river plumes to identify marine areas likely influenced by land-based pollutants. Finally, we prioritised areas for catchment management based on objectives for conservation of terrestrial biodiversity and objectives for water quality that recognised links between pollutant sources and affected marine areas. Our objectives for coastal-marine water quality were to reduce sediment and nutrient discharges from anthropic areas, and minimise future increases in coastal sedimentation and eutrophication. Our objectives for protection of terrestrial biodiversity covered species of vertebrates. We used Marxan, a conservation planning tool, to prioritise interventions and explore spatial differences in priorities for both objectives. Notable differences in the distributions of land values for terrestrial biodiversity and coastal-marine water quality indicated the likely need for trade-offs between catchment management objectives. However, there were priority areas that contributed to both sets of objectives. Our study demonstrates a practical approach to integrating models of catchments, land-use change, and river plumes with conservation planning software to inform prioritisation of catchment management.

## Introduction

Human-induced changes in flows of nutrients and sediments are threatening marine ecosystems [1, 2], compromising the services that oceans provide worldwide [3]. Loads of suspended sediments and nutrients delivered to the oceans have increased drastically following extensive land clearing for cropping, grazing, and coastal development [4, 5]. Eutrophication and sedimentation of marine areas have thus resulted in major impacts on vulnerable coastal ecosystems, such as coral reefs, mangroves, and seagrass [6, 7]. Consequently, there is an urgent need to identify and assess the magnitude of these threats [8], as well as to prioritise management actions to mitigate their impacts on marine ecosystems, particularly in regions where land-based threats are prominent [9, 10].

Alterations to pollutant loads resulting from land-use change have been extensively studied in some regions, such as the Great Barrier Reef in Australia [11] and Chesapeake Bay in the United States [12], with a common finding being a dramatic (up to 10-fold) increase in pollutant discharges to coastal and marine environments. Significant progress has also been made in improving our understanding of the drivers of land-use change and our ability to detect and forecast land-use transitions [13, 14]. Understanding land-use change is critical to quantifying actual changes in pollutant loads (relative to “natural” states) and to setting ecologically-relevant objectives for catchment management [7, 15–17]. Integrating land-use models with catchment models [18] and, importantly, with river-plume models [19, 20] has also proved valuable for assessing the potential extent and impacts of land-based pollutants in the marine environment. Some studies have integrated catchment models with river-plume models [21], and more recently, with land-use change models [22], to guide catchment management. Integrating models of catchments, land-use change, and river-plumes is needed to link potential sources of pollutants within catchments (e.g., sub-catchments, paddocks) to specific marine areas (e.g., highly biodiverse and vulnerable ecosystems), and ultimately to prioritise both terrestrial and marine management actions [23, 24].

Minimising the impacts of land-based threats to marine ecosystems is an important objective of catchment management, but it has to be balanced with other local management objectives, such as protection of terrestrial and freshwater biodiversity, and maintenance of ecosystem services [25, 26]. Consequently, low spatial congruence of parts of catchments important for different objectives (e.g., biodiversity, carbon sequestration, production, water flow regulation) can present managers with difficult trade-offs when allocating land uses and management actions [27–30]. An important step to determine the cost-effectiveness of different management strategies is thus to map the value of parts of catchments for multiple objectives, and use this information to guide the spatial allocation of land uses and actions that maximise the co-benefits and minimise the trade-offs among objectives [25, 29, 31, 32]. However, understanding the spatial congruence between local and downstream values of catchments is incipient [33, 34], and further research is needed to develop methods that can help planners to identify and navigate potential trade-offs arising from competing management objectives [29, 35].

Methods to target catchment management have advanced rapidly in the past decade [15, 17, 36, 37], but integration of these into marine conservation planning is more recent [24, 31, 38]. Over the past 25 years, systematic conservation planning has developed principles, methods, and tools to guide conservation interventions that maximise benefits for biodiversity, while minimising the costs [39, 40]. A number of studies have demonstrated the value of a systematic approach to prioritising catchment management to address land-based threats to marine ecosystems [33, 41, 42], including the exploration of local and downstream conservation benefits [21, 22, 43]. Yet, there is need to improve the quality and accuracy of spatial data and models required for integrated land-sea planning exercises, as well as applications in different contexts, particularly in regions where data are limited [24]. Of particular importance is identifying potential for co-benefits (or trade-offs) between management interventions to achieve local (terrestrial) and downstream (marine) management objectives [27, 44].

Given the need to minimise land-based threats to marine ecosystems and to optimise the use of limited management resources, three key questions arise: **(Q1)** How can we improve targeting of catchment management to reduce end-of-river loads of sediments and nutrients originating from anthropic land uses to maximise benefits to coastal-marine ecosystems?; **(Q2)** How can we identify areas of native vegetation requiring protection to prevent erosion and the delivery of further sediment to marine-coastal areas of conservation importance?; and **(Q3)** Is it possible to protect areas in catchments that contribute to both local (terrestrial) and downstream (marine) management objectives? To answer these questions, we studied the Gulf of California, Mexico, a marine biodiversity hotspot [45] threatened by land-based pollution [46]. A major gap in marine planning globally, including in the Gulf of California [47], is the need to address land-sea connections [23, 24], in particular land-based pollution, which has been identified as a growing threat to marine ecosystems [48].

The overall goal of our study is to develop a method to integrate models of land-use change, pollutant loads from catchments, and river plumes for applications of land-sea conservation planning. The proposed method can help answer our research questions by identifying areas within catchments that need to be protected/managed to maintain or improve coastal-marine water quality (downstream objective) and to conserve terrestrial biodiversity (local objective). Actions within catchments apply to two types of areas: those with native vegetation that could be protected against clearing to minimise further increases in sediment loads and/or to conserve habitat of terrestrial species; and those with anthropic land uses that require management to reduce current loads of pollutants delivered to the sea, for example through the implementation of best-practice management in agriculture. The outputs of our method can also be used to assess the spatial congruence between priority management for water quality and areas that are important for conservation of terrestrial biodiversity, thus helping planners to identify potential co-benefits/trade-offs associated with different management alternatives. Previous studies have found variable congruence between priority areas to achieve downstream (e.g., maintaining water quality/quantity) and local management objectives (e.g., protecting terrestrial ecosystems) [26, 34, 44, 49, 50]; consequently, we expect to find some congruence between priority areas for maintaining water quality and for conserving terrestrial species, but also areas of divergence which can require trade-offs in management decisions.

## Materials and Methods

### 2.1. Study design

**Fig 1:**
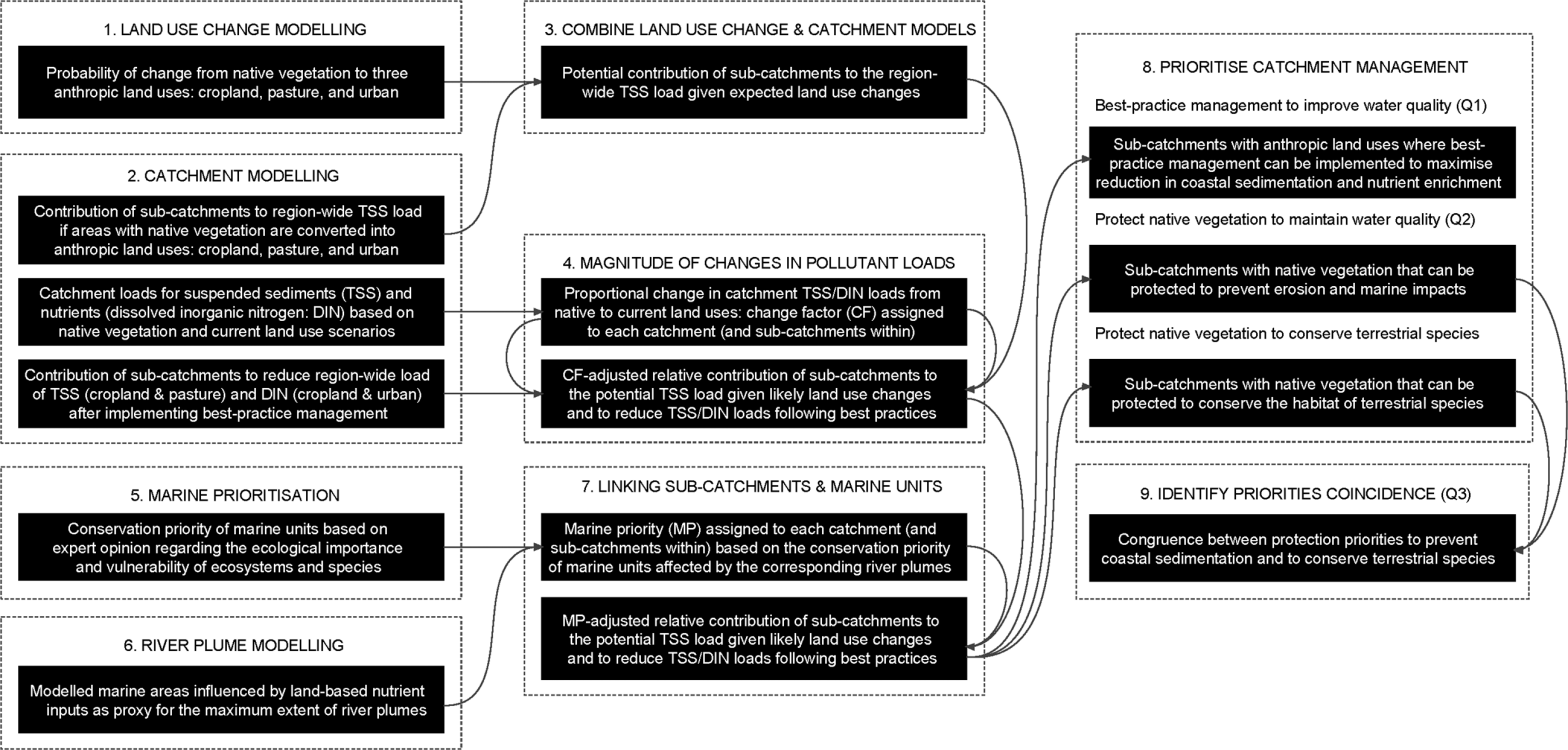
Integration of models and analyses for land-sea planning. Dashed squares represent the models (or broad stages of our method) used to prioritise catchment management to achieve downstream (marine) and local (terrestrial) management objectives. Black boxes depict key outputs of models, as well as derived and integrated outputs resulting from further analyses. Numbers indicate the overall sequence of modelling/analysis and arrows show how outputs are integrated in later stages. Abbreviated question numbers in parenthesis (Q1 to Q3) on the right-hand side of the diagram indicate the final outputs used to answer our three research questions.

Our study proposes a novel method to integrate several models and analyses for land-sea conservation planning. Here we summarise and describe the models used (and key outputs), as well as the integration of models to answer our research questions (**Fig 1**). Numbered statements here refer to numbered parts of Fig 1: (1) We used a land-use change model to estimate the probability of change from native vegetation to anthropic land uses. This is the first step required to identify areas requiring protection to prevent erosion and/or to conserve terrestrial species. (2) We used a catchment model to identify areas prone to erosion if cleared (and thus potentially supplying more suspended sediments to coastal-marine areas) and to estimate potential reductions in pollutant loads (suspended sediment and dissolved inorganic nitrogen) if best-practice management was implemented. (3) We then combined the land-use change and catchment models to estimate the potential erosion resulting from likely changes in land uses. (4) The catchment model was also used to calculate total catchment loads of suspended sediments and dissolved inorganic nitrogen based on two vegetation/land use scenarios (natural and current); these outputs were used to calculate the proportional change in pollutant loads from natural to current conditions to prioritise management of catchments that have experienced larger changes. (5) We used expert opinion to prioritise marine areas based on the ecological importance and vulnerability of marine ecosystems and species within marine management units. (6) We used a plume model to identify the marine areas potentially influenced by pollutants (fine sediment and nitrogen) discharged by rivers. (7) The river plume model was then used to link the land sources of pollutants with affected marine units, thus identifying the sub-catchments to be targeted for management to maximise benefits to marine areas of high conservation priority. (8) We used conservation planning software to identify priorities for improving water quality (preventing erosion and reducing pollutant loads) and for conservation of terrestrial vertebrates. (9) Finally, we compared the priority maps to identify areas of coincidence/divergence between priority maps. See **S1 Fig** for more detail on each part of **Fig 1**.

### 2.2. Study area

**Fig 2:**
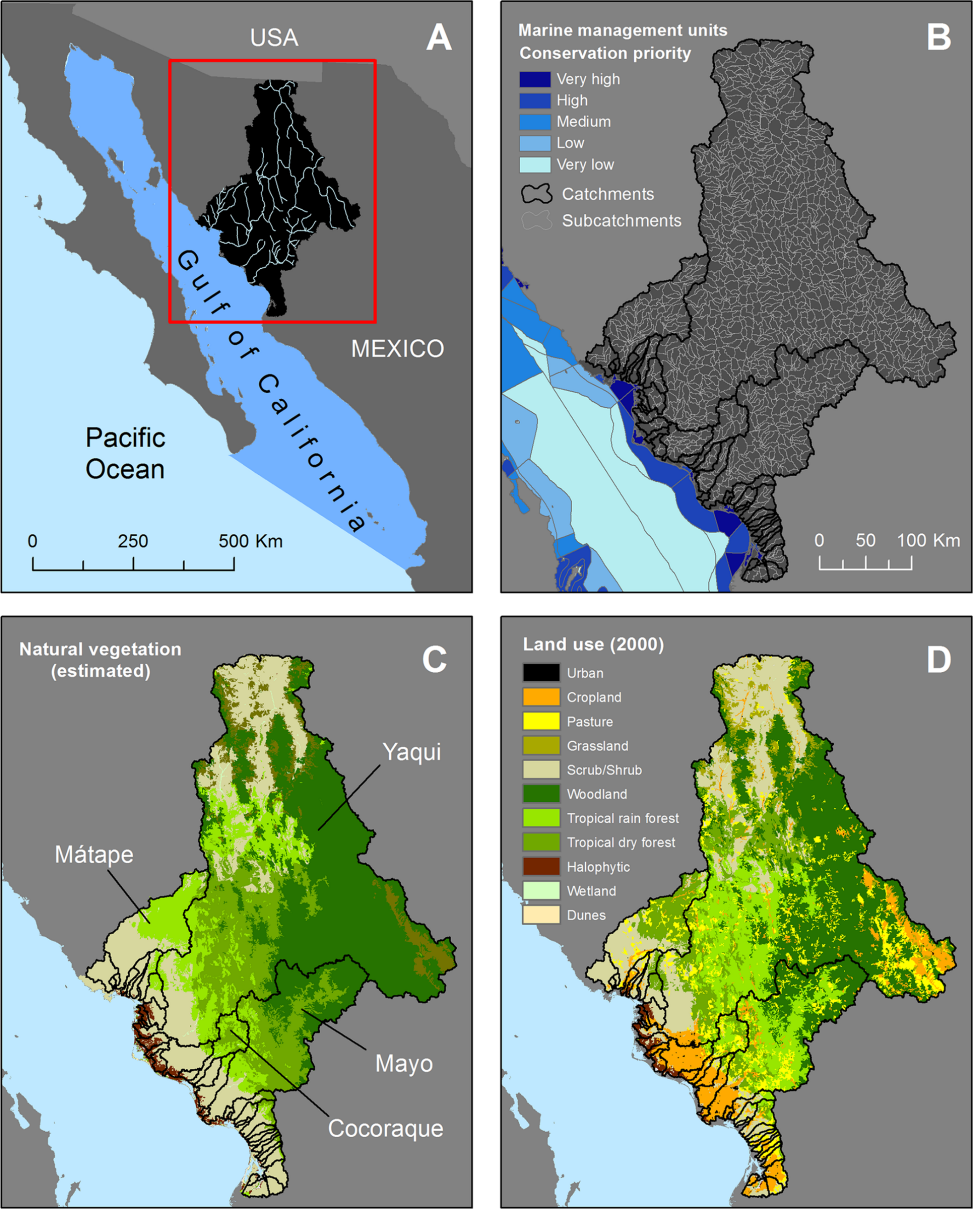
Study area: selected catchments draining into the Gulf of California, Mexico. A) General location of study area and major rivers; B) Catchments, sub-catchments (planning units), and levels of conservation priority of adjacent marine management units; C) Estimated original extent of native vegetation types and largest catchments in the study area; and D) Current (2000) extent of vegetation types and anthropic land uses (cropland, pasture, and urban), reflecting the degree of human modification and underlying the spatial patterns of pollutant supply.

Our study focuses on selected catchments draining into the Gulf of California, Mexico (**Fig 2**). While the western coast of the Gulf of California remains comparatively undisturbed, many eastern coastal areas have been extensively cleared for agricultural and urban land uses [46]. Yet, the region retains large expanses of native vegetation (e.g., woodland, tropical forest and scrub/shrub) and includes priority areas for the conservation of terrestrial biodiversity [51]. The selected catchments are adjacent to marine management units identified as high priorities for conservation of marine ecosystems of regional and national significance [52]. These marine management units are legally recognized and aim to guide the allocation of coastal and marine uses, and management actions; see [52] for further information regarding the regional marine spatial planning exercise defining these units and priorities. A number of coastal and marine areas in the region are affected by land-based pollution [46, 53], sometimes reaching offshore areas [54]. Our study area is thus suitable to explore methods to prioritise the protection and management of catchments to achieve local (terrestrial) and downstream (marine) management objectives, as well as to explore potential trade-offs between these objectives.

### 2.3. Land-use change model

We used a land-use change model to estimate the probability of change from native vegetation to cropland, pasture, and urban areas. Identifying and prioritising the protection of naturally vegetated areas that are more likely to be cleared (for new cropland, pasture, or urban areas) is needed to maximise the potential benefits of management actions [55]. The results of this model were then used to estimate potential increases in pollutant loads associated with clearing of remnant native vegetation (described below); thus we did not model transitions between anthropic land uses (e.g., cropland to urban). This modelling involved three steps: Step 1) exploring and summarising past change, which included correcting land-cover maps and selecting the datasets from the time series to be used in the land-use change model; Step 2) modelling transition potentials from native vegetation to anthropic land-use classes; and Step 3) calculating the probability of change for the modelled transitions within a predefined time period. These analyses were developed using the Geographic Information System (GIS) ArcMap 9.2 [56] and Land Change Modeller (LCM) [57], a tool commonly used for modelling of land-use change [58, 59].

#### Step 1

We explored past changes in land use by comparing three national-scale (1:250,000) land-use/cover (hereafter ‘land use’) datasets: 1976, 1993, and 2000 [60], which were previously corrected for classification inconsistencies arising from different methods to develop them [61]. We used GIS routines and LCM tools to identify and correct remaining class inconsistencies between time-series datasets. This process was needed to identify artificial differences between maps which could result in unlikely or false land-use transitions (e.g., urban to rainforest, rainforest to temperate forest). We selected the 1976 and 2000 datasets as our reference maps to model land-use change because false transitions for this pair of maps were less than those for the other two pairs, i.e., 1976–1993 and 1993–2000 [61]. We also created a map representing ‘native vegetation’, defined as the original extent of native (primary) vegetation before extensive clearing and modern agriculture (**Fig 2C**). The native vegetation map was constructed based on the earliest land-use map available, i.e., 1976 [60], for which anthropic land-use classes were substituted with native vegetation classes using three reference maps: potential vegetation [62], ecoregions of North America [63], and Terrestrial Ecoregions of Mexico [64]. This map was used to estimate the ‘natural’ supply of sediments to identify the proportional increase in loads in comparison to the ‘current’ (2000) land use (**Fig 2D**). Our map of native vegetation should be interpreted with care and used as a proxy in the absence of better information, but further refinements are desirable [65], particularly regarding the definition of appropriate baseline scenarios used to set ecologically relevant targets.

#### Step 2

To model transition potentials, we selected a number of potential explanatory variables (rainfall, dryness, land use, slope, soil type, and proximity to roads and developed areas) to model each of the transition potential sub-models. The selected variables have been identified by a number of studies as important in determining land-use suitability for human occupation and agriculture, or related to physical accessibility [58, 66, 67]. Following current practice, we created three transition potential sub-models, one for each anthropic class (i.e., cropland, pasture, and urban). We selected a set of explanatory variables for each sub-model. Only variables with Cramer’s coefficient V >0.15 for each sub-model were incorporated. Cramer’s statistic has been used previously to test for strength of association (explanatory power) of variables in land-use change applications [18, 68].

#### Step 3

**Fig 3:**
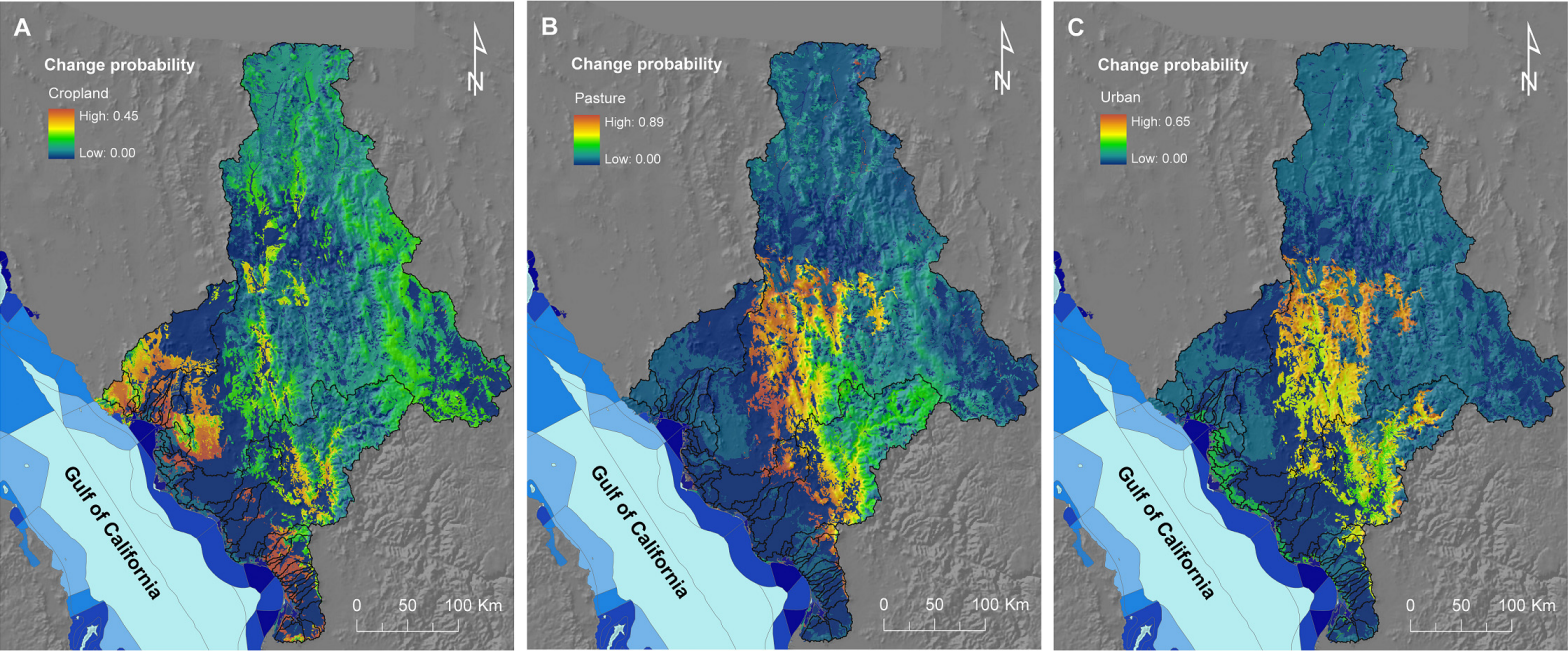
Probability of change from native vegetation classes to anthropic land uses. Maps depict the probability of change from the eight native vegetation classes to A) cropland (***P***_***crops***_), B) pasture (***P***_***pasture***_), and C) urban areas (***P***_***urban***_) for a 20-year period (2000 to 2020), estimated using the land-use change model; also represented are the levels of conservation priority of adjacent marine management units (see legend in **Fig 2B**).

The selected variables were used to calculate the probability of change for the modelled transitions within a 20-year period using a Multi-Layer Perceptron Neural Network [69]. This is a useful technique to model change using multiple explanatory variables simultaneously, and for dealing with non-linear relationships (e.g., gradients in change probability with distance from developed or disturbed areas). We incorporated the influence of two types of conservation areas–protected areas and wildlife management units–in the model as ‘constraints’, thus effectively forcing LCM to reduce the probability of change from natural to anthropic land uses for areas covered by these two management tools. Based on expert advice, we assigned a high-constraint value (0.25) to protected areas [70] and a low-constraint value (0.75) to wildlife management units; these units operate under conservation agreements to protect biodiversity and allow for rational use and management of wildlife [71, 72]. This step resulted in three maps depicting the probability of change from native vegetation classes to each of the three anthropic land uses (**Fig 3**).

### 2.4. Catchment model

We used SedNet [73] to model supply (sub-catchment level) and total load (catchment level) of fine (<63 μm) total suspended sediments (TSS) from hillslope erosion, and nutrients (specifically, dissolved inorganic nitrogen–DIN). We focused on TSS and DIN (typical constituents considered in water-quality modelling and monitoring) because of their potential coastal-marine impacts associated with increased loads [16, 74]. We modelled TSS to identify areas with native vegetation that could be protected against clearing to minimise the increase in sediment loads delivered to coastal-marine areas. We also modelled DIN and TSS exported from cropland, pasture, and urban areas to identify areas that need to be better managed (e.g., through implementation of best-practice management, including fertiliser application and grazing management, [17, 36, 75, 76]) to reduce pollutant loads discharged into the sea. We used SedNet because it is a model appropriate for large-scale applications and was found in this region to predict runoff and suspended sediment loads within their observed ranges of variation [42].

Our main requirements for delineating sub-catchments related to catchment modelling [42] and prioritisation analyses (see ‘**prioritise catchment management: scenarios and objectives**‘ section). We delineated sub-catchments with SedNet by using a drainage area threshold of 40 km^2^, which resulted in 1,655 sub-catchments (later used as planning units for the prioritisation analyses), with a mean size of ~60 km^2^ (standard deviation: 39 km^2^); this ensured that most small coastal catchments were covered by the modelling domain. A detailed explanation of the parameterization of SedNet for this study, as well as a discussion on the advantages, difficulties, and limitations of our catchment model are documented by Álvarez-Romero et al. [42].

We used the catchment model to create three types of outputs: Output 1) total catchment loads for TSS and DIN based on ‘natural’ and ‘current’ land use conditions; Output 2) potential reduction in TSS and DIN supply after implementing best-practice management; and Output 3) maximum TSS supply if existing naturally vegetated areas are transformed into anthropic land uses (which was based on land suitability for cropping, grazing, and urbanization).

#### Output 1

We calculated the total catchment loads (tonne/year) for TSS and DIN by adding the supply of their corresponding sub-catchments, which were modelled using the ‘natural’ and ‘current’ land use maps (**Fig 2C and 2D**). Of the thirty-nine selected catchments draining into the marine management units, four catchments comprise ~88% of the total study area (**Fig 2B**): Yaqui (67,629 km^2^ ≈ 68%), Mayo (13,303 km^2^ ≈13%), Mátape (5,864 km^2^ ≈ 6%), and Cocoraque (1,624 km^2^ ≈ 2%). When combined, these catchments contributed approximately 67% and 87% of the ‘current’ TSS and DIN region-wide loads, respectively [42].

#### Output 2

**Fig 4:**
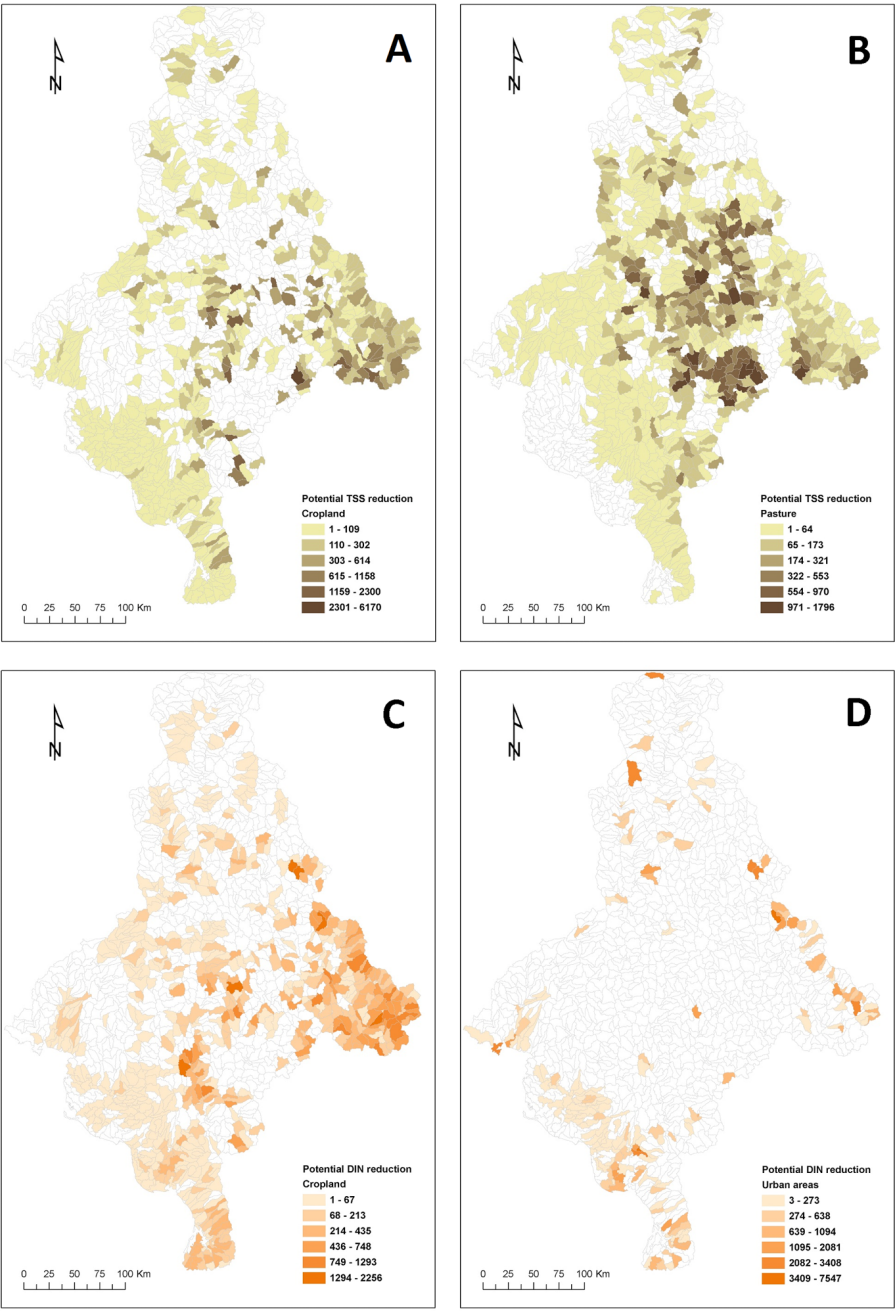
Estimated maximum reduction in the supply of TSS and DIN from anthropic land uses after implementing hypothetical best-practice management. Maps show the maximum reduction (kg/year), at sub-catchment level, in total suspended sediment (TSS) supply for A) cropland and B) pasture; and in dissolved inorganic nitrogen (DIN) for C) cropland and D) urban areas. Only sub-catchments including anthropic land uses were targeted for management in the prioritisation analyses based on best-practice management scenarios.

To create the scenarios of potential reduction in TSS and DIN supply from implementing best-practice management we followed two steps. First, we created maps representing the estimated supply from sub-catchments of DIN and TSS if 100% of the areas under cropland, pasture, and urban areas implemented best-practice management to reduce these two pollutants. To simulate reductions in DIN supply associated with best-practice management, we adjusted the event mean concentration–EMC [77] of DIN for cropland and urban areas (**Table 1**). These values were set to represent realistic potential reductions in nutrient runoff based on reported reductions from literature [78, 79]. Since potential reductions are higher for areas where fertiliser use is more intense, we modified the EMC values according to cropland classes, adjusting it based on their relative use of fertiliser (i.e., very high use: 40% reduction; high use: 30% reduction; moderate use: 20% reduction; low use: 10% reduction; very low use: 5% reduction). For urban areas, the revised EMC value corresponds to a maximum 20% reduction. We excluded pasture from the DIN reduction scenario because pasture management practices have a minor impact on DIN concentrations. We simulated the effects of implementing best-practice managements to reduce TSS supply by modifying the input into the catchment model known as the cover factor (C-factor); the C-factor was used in the Revised Universal Soil Loss Equation (RUSLE) to calculate soil loss [80] for cropland and pasture [42]. We assumed a maximum potential TSS reduction of 30% from cropland and 20% from pasture, respectively (**Table 1**). We excluded urban areas from the TSS reduction scenario because, once these are established, they do not contribute significantly to erosion. Second, we calculated the difference between the ‘best-practice management’ and ‘current’ scenarios (described above) to represent a hypothetical maximum reduction (**Fig 4**).

**Table 1:** Nutrient event mean concentrations (EMC) and cover factor (C-factor) values (for DIN and TSS, respectively) used for catchment modelling. The first set of values corresponds to the original EMC (mg/L) and C-factor (non-dimensional parameter) values used to model current and maximum supply scenarios. Numbers in parentheses and bold are the modified parameters used to simulate TSS and DIN reductions resulting from implementing best-practice management. We classified cropland areas based on their relative use of fertiliser, from very low to very high [81] and used this classification to progressively assign EMC values for each class (lowest to highest) using the 50^th^, 60^th^, 70^th^, 80^th^, and 90^th^ percentiles of documented values for cropland (see **S1 Text**).

Anthropic land uses	Dissolved inorganic nitrogen (DIN)–EMC	Total suspended sediment (TSS)–C-factor
Urban areas	0.794 (**0.635**)	0.005 (**Not modified**)
Cropland (5 classes)	Very high.…1.500 (**0.900**)	0.261 (**0.183**)
	High….…. . .1.047 (**0.733**)	
	Moderate. . . .0.850 (**0.680**)	
	Low……. . . .0.750 (**0.675**)	
	Very low….0.700 (**0.665**)	
Pasture	0.399 (**Not modified**)	0.230 (**0.184**)

#### Output 3

Finally, we modelled the maximum TSS supply assuming all areas with remnant native vegetation are transformed into anthropic land uses. We created these maps by applying a uniform C-factor (one for each of the modelled anthropic land uses: cropland, pasture, or urban areas) to all areas with any of the eight native vegetation classes. The resulting TSS supply maps were then adjusted using the results of the land-use change model (details below), effectively reducing the maximum TSS supply of a given area based on its probability of being converted from native vegetation to anthropic land uses.

### 2.5. Combining land-use change and catchment models

**Fig 5:**
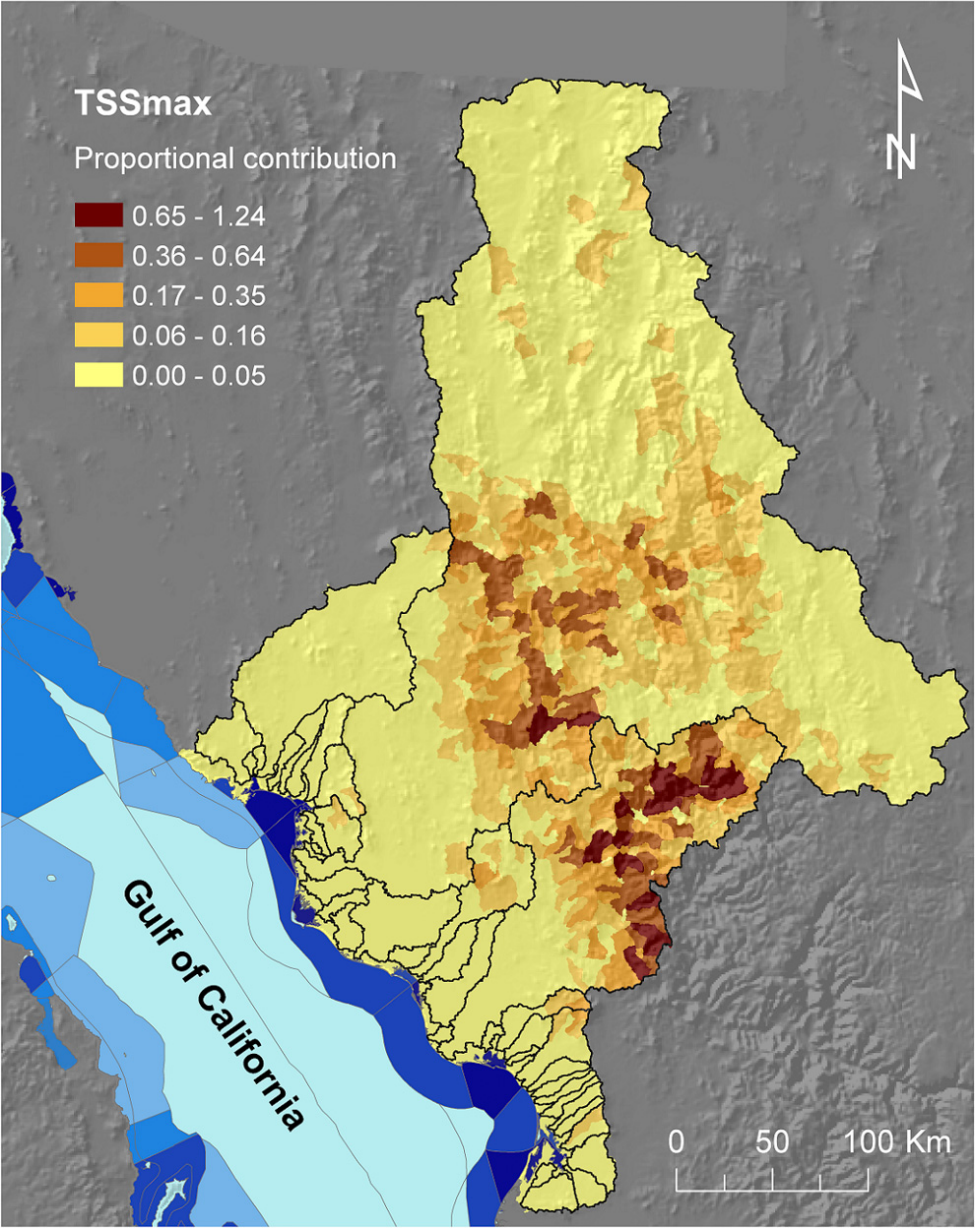
Maximum proportional contribution (percentage) of sub-catchments to the region-wide TSS load, considering the potential conversion of native vegetation to anthropic areas. The map integrates the individual maps depicting the potential supply of total suspended sediment (TSS) if natural vegetated areas are converted into anthropic land uses (i.e., cropland, pasture, or urban areas), each previously multiplied by the probability of change from native vegetation to the corresponding anthropic land use (**Fig 3**); also represented are the levels of conservation priority of adjacent marine management units (see legend in **Fig 2B**).

The outputs from the land-use change and catchment models were combined to create three maps representing the estimated proportional contribution of sub-catchments to the region-wide TSS load given the probability of change from native vegetation into each of the three anthropic land uses. We focused on TSS because we modelled only probability of change (i.e., soft prediction) to anthropic land uses and not the future distribution of land uses (i.e., hard prediction), which would be required to model increases in DIN loads. To do this, we began with the maps showing the estimated maximum TSS supply, assuming all areas with native vegetation are converted into cropland, pasture, or urban areas, and transformed them into maps depicting the proportional contribution (%) of each sub-catchment (only sub-catchments with native vegetation) to the region-wide (study area) TSS load. Then, we combined each of the transformed maps (i.e., proportional maximum contribution from each transition, including natural-to-cropland: ***TSS***_***crops_max***_, natural-to-pasture: ***TSS***_***pasture_max***_, and natural-to-urban: ***TSS***_***urban_max***_) with the maps depicting the probability of change from native vegetation to anthropic land uses (one per transition: natural-to-cropland: ***P***_***crops***_, natural-to-pasture: ***P***_***pasture***_, and natural-to-urban: ***P***_***urban***_, **Fig 3**) calculated using the land-use change model (see ‘**land-use change model**‘ section). Finally, we used the following function (Eq 1) to integrate the three combined maps (each depicting proportional TSS contribution adjusted by the probability of change into the corresponding anthropic land use) into a map representing the integrated maximum proportional contribution (***TSS***_***max***_); this map (**Fig 5**) represents the proportional contribution of sub-catchments to the region-wide TSS load given expected land use changes:

$${\mathrm{Tss}}_{\max}=\left(P_{\mathrm{crops}}\times {\mathrm{TSS}}_{\mathrm{crops}\_\max}\right)+\left(P_{\mathrm{pasture}}\times {\mathrm{TSS}}_{\mathrm{pasture}\_\max}\right)+\left(P_{\mathrm{urban}}\times {\mathrm{TSS}}_{\mathrm{urban}\_\max}\right)$$ (1)

### 2.6. Magnitude of changes in end-of-river loads

**Fig 6:**
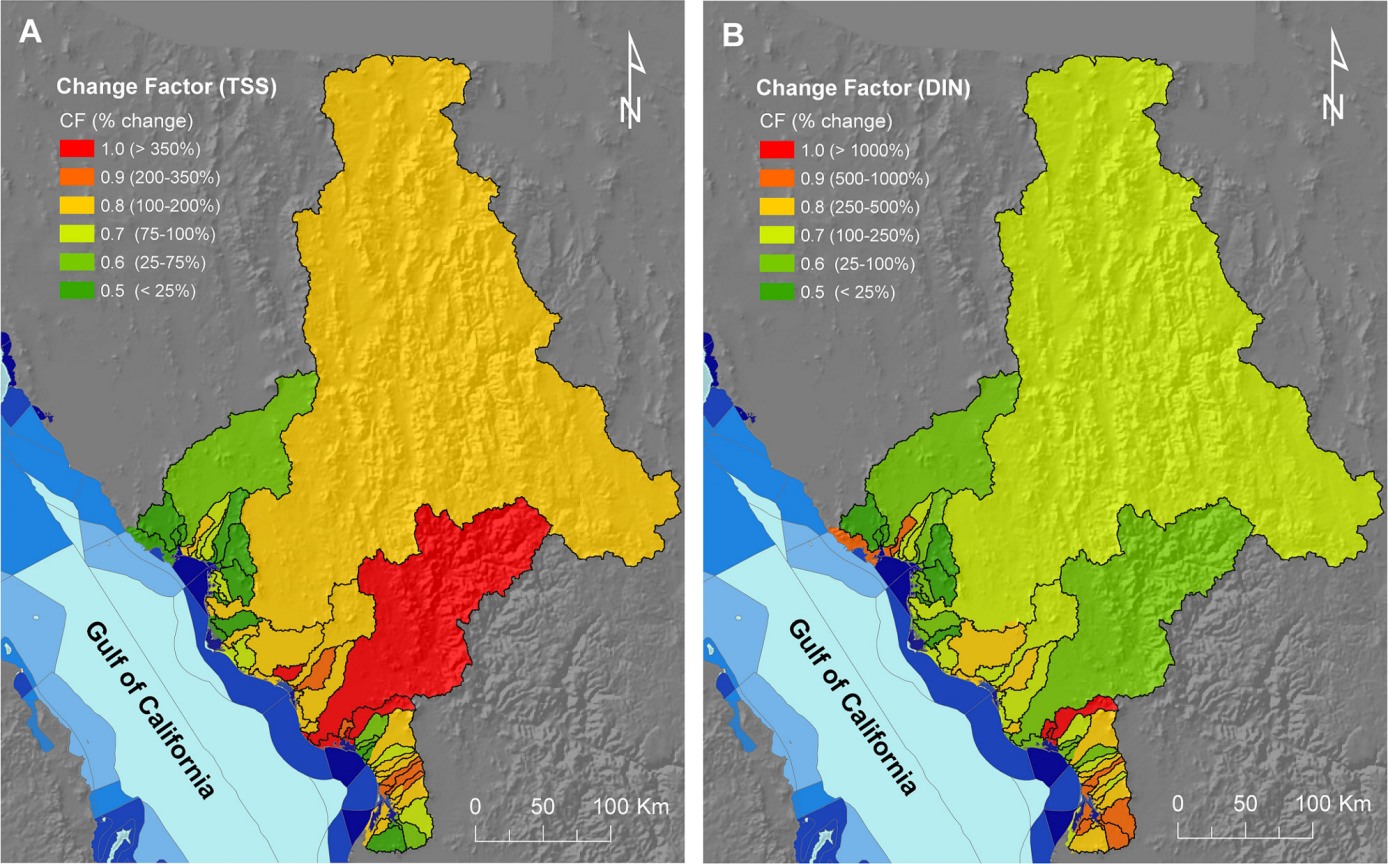
Change factors of the thirty-nine catchments in our study area. Values for change factor (***CF***) were allocated according to the estimated change in (A) total suspended sediment (TSS) and (B) dissolved inorganic nitrogen (DIN) loads from the ‘natural’ (**Fig 2C**) to the ‘current’ (**Fig 2D**) vegetation/land use conditions. Also represented are the levels of conservation priority of adjacent marine management units (see legend in **Fig 2B**).

Quantifying changes in pollutant loads (relative to “natural” states) is necessary to set ecologically-relevant objectives for catchment management [7, 17]. This information can help to understand observed (or potential) effects of increased pollutant loads on marine ecosystems and adjust management accordingly [15, 16]. However, empirical information about responses of marine ecosystems is lacking for many regions (including the Gulf of California), thus limiting our ability to identify the required reductions in pollutant loads. Given the limited information about ecological responses of marine ecosystems to current pollutant loads, we assumed that catchments that have experienced larger changes in the amount of exported sediments and nutrients are, at least potentially, having a higher impact on marine ecosystems (i.e., all else being equal, catchments of lower natural integrity should be prioritised for management). Consequently, we further adjusted the maps representing potential TSS/DIN reduction (**Fig 4**) and TSS supply (**Fig 5**) to incorporate the magnitude of these changes. To do this, we calculated a change factor (**CF**), representing the proportional change in TSS/DIN load from ‘natural’ to ‘current’ land use conditions, which was calculated at catchment level (**Fig 6**). We assigned a ***CF*** to each catchment (and sub-catchments within them) based on the observed proportional changes in loads of TSS and DIN from ‘natural’ to ‘current’ land use according to **Table 2**. Effectively, this reduced the importance (in terms of proportional contribution) of sub-catchments that have experienced less change in the amount of exported sediments and nutrients; see ‘**linking sub-catchments with marine units**‘ section.

**Table 2:** Ranges of proportional change in catchment loads of pollutants (DIN and TSS) from ‘natural’ to ‘current’ land-use conditions used to calculate the change factor (*CF*). We arbitrarily applied progressively larger ***CF*** values, from 0.5 (effectively reducing the importance of the catchment by half) when the estimated increase in loads of DIN or TSS was relatively low (<25%) to 1.0 (maximum importance) for catchments with very large increases in estimated loads of pollutants (>1,000% for DIN and >350% for TSS). Due to high variability in the proportional change in pollutant loads between catchments and pollutants, we assigned different intervals for ***CF*** categories for TSS and DIN, in both cases based on a geometric increase, which fitted the distribution of our data.

Dissolved inorganic nitrogen (DIN)	Suspended sediment (TSS)	Change Factor (*CF*)
< 25%	< 25%	0.5
25–100%	25–75%	0.6
100–250%	75–100%	0.7
250–500%	100–200%	0.8
500–1000%	200–350%	0.9
> 1,000%	> 350%	1.0

### 2.7. Marine priorities

This step was needed to prioritise catchments (for downstream benefits) based on the conservation priority of marine units that are likely influenced by their corresponding river plumes. We used the model of Halpern et al. [82], in combination with the priority level of marine management units, **Fig 2B** [52], to assign a marine priority (***MP***) factor to associated catchments; see ‘**linking sub-catchments with marine units**‘ section. The ***MP*** factor represents the importance of sub-catchments for coastal-marine water quality based on the priority of marine management areas that are potentially affected by the catchment to which each sub-catchment belongs. Therefore, sub-catchments that are linked to marine units with high conservation priority (i.e., areas considered by federal and state governments as a priority for conservation or management) have a higher ***MP*** value. The ***MP*** was calculated using the following function based on expert opinion on the ecological importance and vulnerability of ecosystems and species within marine management units (Eq 2):

MP=CI×VI (2)

Where

***CI***: Conservation Index (0 to 1): ecological importance based on species richness, productivity, presence of threatened/protected species (e.g., *Phocoena sinus*, *Totoaba macdonaldii*, sea turtles, cetaceans, sharks), endemic algae, wetlands, bays and coastal lagoons, and protected areas.

***VI***: Vulnerability Index (0 to 1): higher values indicate areas where high fragility of coastal-marine ecosystems and human-pressures (other than catchment effects) co-occur.

Both indices (***CI*** and ***VI***) were allocated to marine management units following a consultative process with experts and stakeholders as part of a marine spatial planning process for the Gulf of California [52].

### 2.8. River-plume model

**Fig 7:**
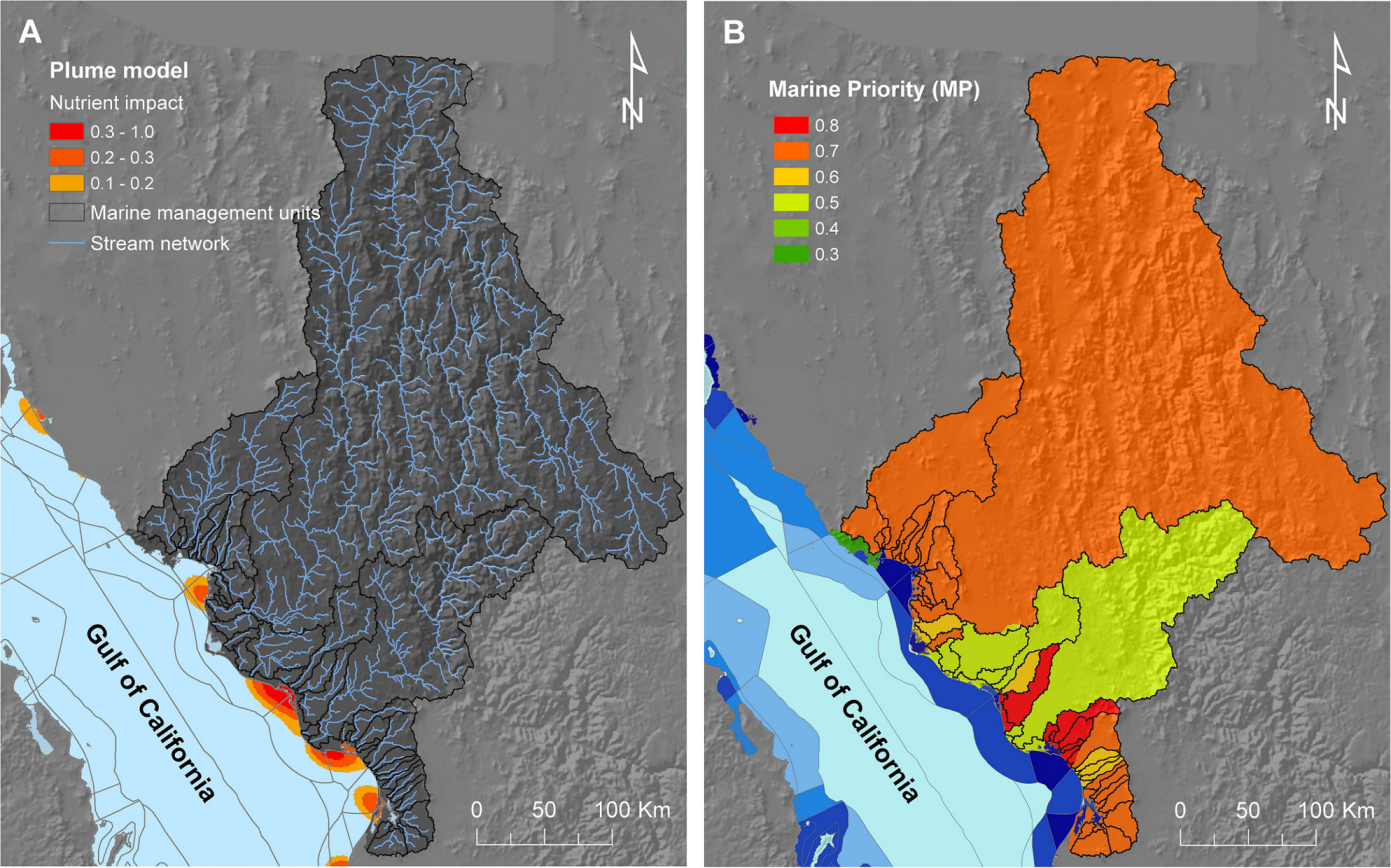
River-plume model and priority level of catchments for coastal-marine conservation. A) River-plume model depicting potential impacts from land-based nutrient pollution [82] used as a proxy for the maximum extent of influence of rivers. B) Marine priority (***MP***) factor assigned to each catchment (and subsequently to the sub-catchments within them), representing their importance for marine conservation in terms of their link to marine management units of varying conservation priority (see legend in **Fig 2B**).

We used a global model for land-based threats [82] as an approximation of river plumes in the study area. As part of their map of cumulative land-based impacts, Halpern et al. [82] modelled marine areas likely influenced by nutrient inputs, which we used as a proxy for the maximum extent of influence of river plumes (**Fig 7A**). We identified the catchments influencing marine areas by overlaying mapped river plumes (modelled by Halpern et al. [82]) and marine management units. Catchments (and sub-catchments within them) were thus linked with the marine management areas affected by their corresponding river plumes. Where catchments affected more than one marine management unit, we followed a conservative approach and used the link to the marine unit with highest ***MP*** value (**Fig 7B**). Due to the lack of spatial information on the extent of river plumes originating from the smaller coastal catchments, we assumed that their influence would be restricted to marine management units into which they drain directly. Based on the link to and priority of marine management units (**Fig 2B**), we adjusted the pollutant supply maps (**Figs 4 and 5**); see ‘**linking sub-catchments with marine units**‘ section. The adjusted maps were then used to prioritise the protection or management of sub-catchments to achieve our marine objectives (i.e., for water quality).

### 2.9. Linking sub-catchments with marine units

We used the following functions (Eqs 3–6) to incorporate the link between sub-catchments and marine areas, through scaling the four maps depicting maximum potential reduction in TSS/DIN following management of anthropic areas (i.e., ***TSS***_***crops-reduction***_, ***TSS***_***pasture_reduction***_, ***DIN***_***crops_reduction***_, ***DIN***_***urban_reduction***_, **Fig 4**) using the two factors described above: change factor (***CF***: **Fig 6**) and marine priority (***MP***: **Fig 7B**):

$${\mathrm{TSS}}_{\mathrm{crops}\_\mathrm{scaled}}={\mathrm{TSS}}_{\mathrm{crops}\_\mathrm{reduction}}\times {\mathrm{CF}}_{\mathrm{TSS}}\times \mathrm{MP}$$ (3)

$${\mathrm{TSS}}_{\mathrm{pasture}\_\mathrm{scaled}}={\mathrm{TSS}}_{\mathrm{pasture}\_\mathrm{reduction}}\times {\mathrm{CF}}_{\mathrm{TSS}}\times \mathrm{MP}$$ (4)

$${\mathrm{DIN}}_{\mathrm{crops}\_\mathrm{scaled}}={\mathrm{DIN}}_{\mathrm{crops}\_\mathrm{reduction}}\times {\mathrm{CF}}_{\mathrm{DIN}}\times \mathrm{MP}$$ (5)

$${\mathrm{DIN}}_{\mathrm{urban}\_\mathrm{scaled}}={\mathrm{DIN}}_{\mathrm{urban}\_\mathrm{reduction}}\times {\mathrm{CF}}_{\mathrm{DIN}}\times \mathrm{MP}$$ (6)

As for the previous functions (regarding management of anthropic areas), we used the following function (Eq 7) to incorporate the link to marine areas in the maps used to prioritise protection of native vegetation through scaling the modelled maximum proportional contribution of TSS of each sub-catchment (***TSS***_***max***_, **Fig 5**) using the proportional change (***CF***: **Fig 6A**) and marine priority (***MP***: **Fig 7B**) factors:

$${\mathrm{TSS}}_{\mathrm{scaled}}={\mathrm{TSS}}_{\max}\times {\mathrm{CF}}_{\mathrm{TSS}}\times \mathrm{MP}$$ (7)

In both sets of functions (above), the ***CF*** factor was incorporated to increase the importance of sub-catchments that have experienced larger changes in the amount of exported sediments and nutrients and are, at least potentially, having a higher impact on marine ecosystems. Likewise, the ***MP*** factor adjusts the importance of sub-catchments for coastal-marine water quality based on the priority of marine management areas that are potentially affected by the catchment to which each sub-catchment belongs. The scaled TSS/DIN maps were thus used to represent the relative contribution of sub-catchments to achieving water quality objectives (described below).

### 2.10. Prioritise catchment management: scenarios and objectives

We defined three management scenarios to guide our prioritisation: Scenario 1 (**S1**)–implementation of best-practice management in anthropic areas to improve coastal-marine water quality; Scenario 2 (**S2**)–protection of native vegetation to maintain coastal-marine water quality; and Scenario 3 (**S3**)–protection of native vegetation to conserve terrestrial biodiversity (**Table 3**). Scenarios 1 and 2 incorporate objectives to minimise land-based pollution to marine areas of conservation priority. We used the decision-support tool Marxan [83] to explore our management scenarios, with sub-catchments (delineated using SedNet) as our planning units. We set the costs of planning units equal to their areas because the costs of specific management actions could not be defined accurately using available information. Our scenarios were designed to inform variable types of management (from improving fertiliser use to implementing protected areas), each with different costs and distributions of costs to stakeholders (e.g., protected land might include government acquisition or private funding of management agreements). Below we describe the scenarios and how we defined features and objectives for use in Marxan; additional details about parameterisation of Marxan are given in the caption for **Table 3**.

**Table 3:** Summary of parameterisation for Marxan prioritisation scenarios. For each scenario we used the area of planning units (sub-catchments) as cost (multiplied by 100). We assigned a baseline feature penalty factor (FPF) of 10. Clumping of planning units was not required given the size of sub-catchments and the types of management actions considered; thus we set the boundary length modifier (BLM) to zero. Each scenario was run 100 times, with 1,000,000 iterations each.

	Scenarios	Description	Features targeted	Objectives
*Management of anthropic areas*	**S1**. Improve coastal-marine water quality through best-practice management in anthropic areas	Implement best-practice management in areas with anthropic land uses to reduce loads of nitrogen and sediment delivered to priority marine conservation areas	Modelled maximum reduction of DIN (from cropland and urban areas) and TSS (from cropland and pasture), scaled using the change factor (***CF***) and marine priority (***MP***)	Arbitrarily set as 30% of the scaled TSS and DIN maximum potential reduction from the corresponding anthropic land use
*Protection of naturalvegetation*	**S2**. Maintain coastal-marine water quality through the protection of native vegetation	Protect areas with native vegetation that are prone to erosion and likely to be converted into anthropic land uses to avoid increases in sediment delivered to priority marine conservation areas	Modelled maximum supply of TSS from sub-catchments with remnant native vegetation, scaled using the change factor (***CF***) and marine priority (***MP***)	Arbitrarily set as 30% of the scaled maximum TSS supply from natural areas if converted to anthropic land uses
	**S3**. Conserve terrestrial biodiversity through protection of native vegetation	Protect areas with remnant native vegetation that are important for the conservation of vertebrate species of conservation concern	Modelled distributionsof endangered and protected species of terrestrial vertebrates	Set as variable percentages of potential distributions; objective for each species determined following expert opinion

#### S1) Implement best-practice management in anthropic areas to improve water quality

The first set of water-quality objectives considered the implementation of best-practice management in sub-catchments with anthropic land uses (cropland, pasture, and urban areas) to reduce loads of DIN and TSS delivered to high-priority marine conservation areas (**Table 3**). We set four objectives (two for TSS: cropland and pasture and two for DIN: cropland and urban areas) based on potential reductions in end-of-river loads of TSS and DIN following the implementation of best-practice management (see ‘**catchment model**‘ section). In the absence of information to determine ecologically based objectives (e.g., considering responses of marine ecosystems to pollutant levels), we arbitrarily set our objectives as 30% of the scaled maximum potential reduction of TSS (for cropland and pasture) and DIN (for cropland and urban areas) following implementation of best-practice management. Our water quality objectives are comparable to recent target-setting exercises, which take into account technical and socioeconomic feasibility [16, 84].

#### S2) Protect native vegetation to maintain coastal-marine water quality

The second water-quality objective concerned maintenance of end-of-river water quality through the protection of sub-catchments with remnant native vegetation to prevent erosion and thus sedimentation impacts on coastal-marine ecosystems (**Table 3**). This objective aimed to avoid as much additional loss of water quality as possible, by prioritising the protection of sub-catchments with both the highest risk of transformation and the highest potential contribution to end-of-river TSS loads if transformed. As above, we arbitrarily set this objective as 30% of the scaled maximum TSS supply from sub-catchments with remnant native vegetation that could potentially contribute to the region-wide TSS load if converted into anthropic land uses.

#### S3) Protect native vegetation to conserve terrestrial vertebrates

The second objective regarding protection of native vegetation was to protect natural areas to conserve terrestrial biodiversity (**Table 3**), in this case vertebrate species (47 mammals, 80 birds, 43 reptiles, and 16 amphibians). Variable objectives were set for different species, ranging from 5% to 100% of their potential distributions. We used the objectives that the Mexican Government determined for species based on their conservation and protection status following expert advice [85]. We used expert-validated modelled species distributions (at 1 km resolution) generated using the Genetic Algorithm for Rule-set Production (GARP) tool [86] for: mammals [87], birds [88], amphibians [89], and reptiles [89].

### 2.11. Identify congruence between priority maps for different objectives

For each scenario, our maps of land values at the resolution of planning units (sub-catchments) were the ‘summed solution’ (hereafter ‘selection frequency’) and ‘best solution’ from Marxan analyses. The selection frequency shows the number of times, out of 100 Marxan solutions, that each planning unit was selected. The best solution shows one possible configuration of sub-catchments that achieves the defined objectives at least cost, i.e., the most efficient solution of the 100 Marxan runs [90]. We compared the summed- and best-solution maps from the three scenarios to identify the areas of coincidence (comparable frequencies and congruence between best solutions) and areas of divergence (different frequencies, indicating potential trade-offs between objectives). For the scenarios regarding protection of vegetation (i.e., terrestrial biodiversity and water quality for TSS erosion) we created a map depicting the differences in selection frequencies of planning units (i.e., subtracting the selection frequency map of **S3** from that of **S2**).We also created a map identifying the areas of coincidence in selection frequency (i.e., the number of times that each unit was selected in both **S2** and **S3**) to identify areas where protection of native vegetation scenarios could lead to co-benefits for water quality and biodiversity conservation.

## Results

**Fig 8:**
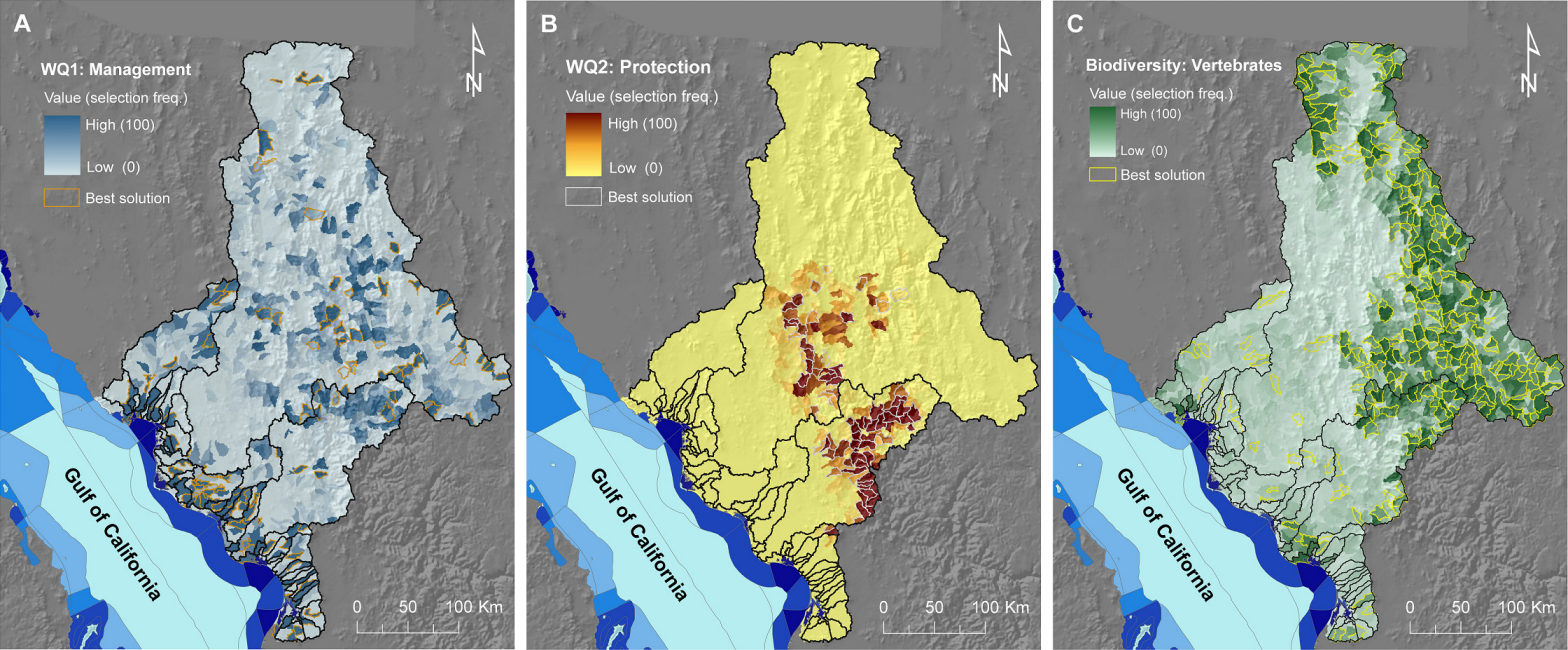
Priorities for catchment management and protection. Maps show the relative value or priority of sub-catchments for achieving two sets of marine objectives (improving or maintaining end-of-river water quality) and one set of terrestrial objectives (conservation of terrestrial vertebrates). The values of planning units are represented by the selection frequency maps of Marxan. Sub-catchments selected more frequently in Marxan runs (darker colours) indicate their higher importance for achieving objectives. Also shown in the maps are the Marxan best solutions for each scenario. A) **S1**: Priorities for management to improve coastal-marine water quality by implementing best-practice management in anthropic land uses (cropland, pasture, and urban); B) **S2**: Priorities to maintain water quality through protection of remnant native vegetation to minimise the increase in sediment loads delivered to coastal-marine areas; and C) **S3**: Priorities for conservation of terrestrial vertebrate species; also represented is the level of conservation priority of adjacent marine management units (see legend in **Fig 2B**).

The Marxan results for the water quality scenarios (**S1** and **S2**) followed expected patterns. The selection frequency map for best-practice management (**S1**) was spatially sparse (**Fig 8A**), which can be partially related to the patchy distribution of anthropic land uses (**Fig 2D**), but also to the divergent distribution patterns of areas targeted for the reduction in TSS or DIN loads (**Fig 4**). In contrast, the selection frequency map for the protection water-quality objective (**S2**) was notably clumped within the two larger catchments (**Fig 8B**), with higher selection frequencies corresponding with sub-catchments potentially contributing the most to the TSS load, **Fig 5**).

Our results showed important differences in the spatial distribution of local (terrestrial) and downstream (coastal-marine) land values, for both the management (**S1**: **Fig 8A**) and protection (**S2: Fig 8B** and **S3**: **8C**) scenarios. Differences between the selection frequency maps for achieving water-quality objectives through management (**S1**) and protection (**S2**) were particularly striking, showing a negative correlation (Spearman’s rank correlation coefficient: -0.414, *p* < 0.0001). However, these were expected because selections for management objectives targeted sub-catchments containing anthropic land uses to improve water quality (**Figs 2 and 4**) whereas selections for protection targeted sub-catchments with remnant native vegetation, particularly those areas with potential for both high erosion and extensive clearing (**Fig 5**). Consequently, a comparison of the best-solution outputs from Marxan for the two water quality scenarios showed almost no overlap (only two sub-catchments; not shown), indicating that sub-catchments with both high current supply of TSS and DIN (valuable for management) and sub-catchments with significant remnant native vegetation and high erosion potential (valuable for protection) were rare. The configurations of the best-solution outputs for the management and protection scenarios also differed notably. For instance, the best solution for the management scenario (**S1**) contained 134 sub-catchments (8.1% of total number) and covered 8,600 km^2^ (8.4% of the study area). Conversely, the best solution for the protection scenario (**S2**) contained only 67 sub-catchments (4.1% of total number) and covered 5,880 km^2^ (5.8% of the study area).

**Fig 9:**
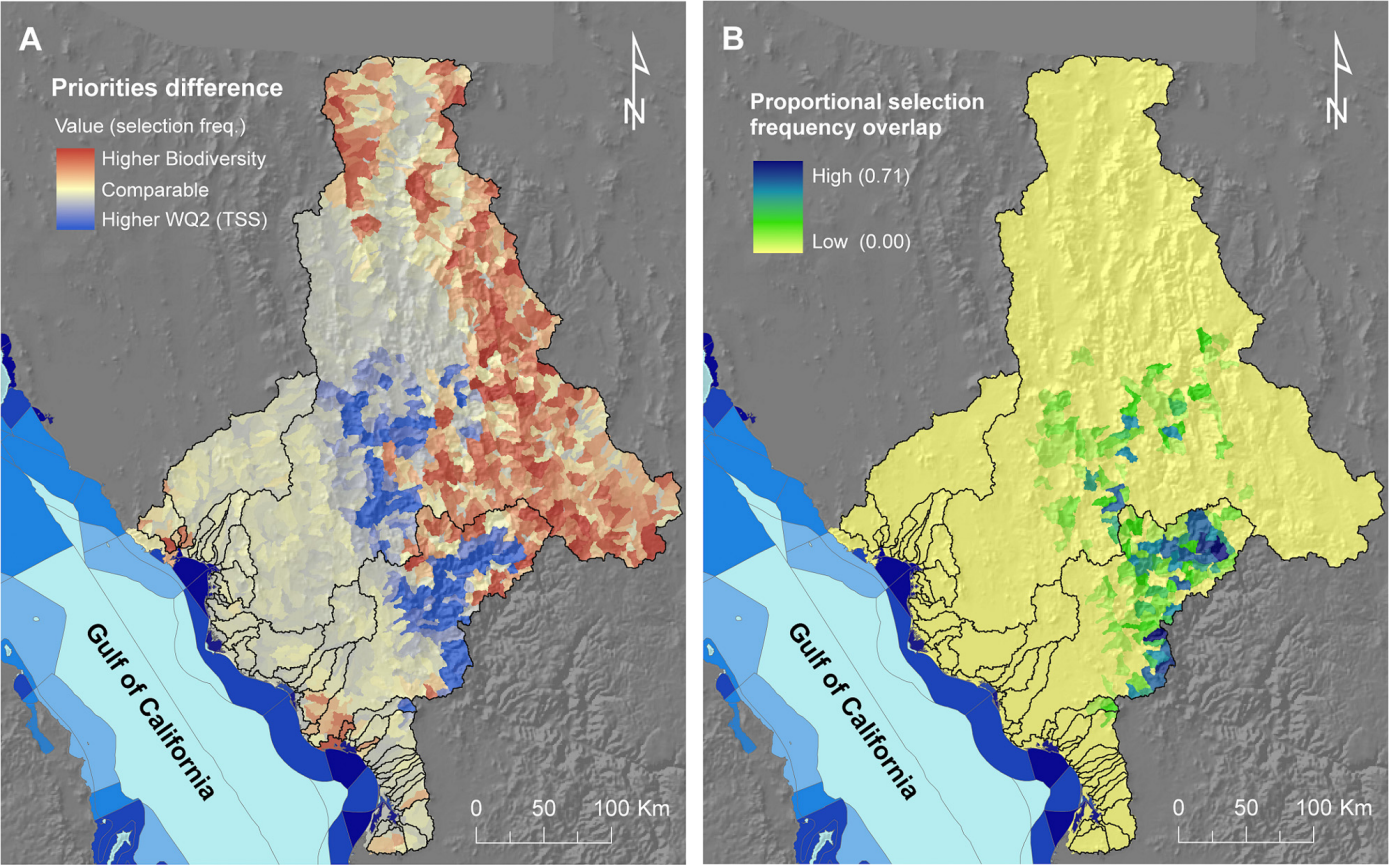
Spatial similarities and differences between priority maps for water quality protection and protection of terrestrial biodiversity. A) Differences between the two maps calculated by subtracting the selection frequency map of **S3** (terrestrial biodiversity) from that of **S2** (water quality): red areas were preferentially selected for terrestrial biodiversity, while blue areas were selected more often for protection against erosion. Areas in beige and light red or light blue had comparable selection frequencies for the two scenarios (either both low or both high, but most of these areas depict areas of low selection frequencies in both scenarios). B) Areas of coincidence, measured as the number of times (out of 100 runs) units were selected in both scenarios; also represented is the level of conservation priority of adjacent marine management units (see legend in **Fig 2B**).

Higher selection frequencies for terrestrial vertebrates (**S3**) were concentrated in the upper regions of the Yaqui and Mayo catchments (**Fig 8C**). Overall, there was very low spatial similarity between the selection-frequency maps for protection to maintain water quality (**S2**: **Fig 8B**) and that for protecting vertebrate species (**S3**: **Fig 8C**), with a weak negative relationship overall (Spearman’s rank correlation coefficient: -0.177, *p* < 0.0001). The number of units and total area of the best-solution output for terrestrial vertebrates was much larger (i.e., 244 units, 14.8% of total number, and covering 21,611 km^2^, 21.2% of the study area) than in the best solutions for the other two scenarios. A visual comparison of the maps for protection for water quality and protection for vertebrates (**Fig 8B and 8C**) revealed that few sub-catchments were part of the best solutions in both scenarios. Subtracting the selection frequency map of **S3** from **S2** highlighted these differences (**Fig 9A**). However, we found some coincidence for a number of sub-catchments (represented as areas of high coincidence in **Fig 9B**), indicating some potential to achieve both water quality and biodiversity objectives through the protection of these areas.

## Discussion

### How can we improve targeting of catchment management to reduce end-of-river loads of sediments and nutrients originating from anthropic land uses to maximise benefits to coastal-marine ecosystems?

The method proposed here can help catchment managers to identify sub-catchments with anthropic land uses where best-practice management can be implemented to achieve, with a budget, the maximum potential reduction in coastal sedimentation and nutrient enrichment. Using the proportional change in catchment TSS/DIN loads from natural to current land uses (***CF***) to adjust the potential contribution of sub-catchments to reduce TSS/DIN loads allowed us to prioritise the management of catchments that are (potentially) having larger effects on marine ecosystems. Likewise, including the priority of marine management units further directed efforts to sub-catchments linked to marine areas of higher conservation priority. A limitation of this management scenario is the lack of information to set ecologically relevant objectives based on potential responses of ecosystems and species to current and improved water quality [15–17]. Ideally, identifying ecologically-informed objectives would involve three steps, working back from affected marine ecosystems to parts of catchments. First, the water-quality standards appropriate to maintaining the structure and composition of marine ecosystems would be informed by expert opinion [6], incorporating where possible data on the effects of pollutants on particular ecosystems and species. Second, a river plume exposure model would identify potential impacts of catchments on marine ecosystems [91] and inform end-of-river objectives for water quality. Third, spatially explicit catchment models would identify the management and protection within sub-catchments needed to meet the end-of-river objectives.

### How can we identify areas of native vegetation requiring protection to prevent erosion and the delivery of further sediment to marine-coastal areas of conservation importance?

A critical step in prioritising the protection of natural areas is determining the likelihood of these areas being cleared [55]. Using a land-use change model allowed us to identify areas with a high probability of being transformed into different anthropic land uses; this information was needed to estimate the potential increases in pollutant loads associated with different land uses. Following the steps outlined in the previous section, the integration of the catchment and river-plume models, and linking of marine areas to sub-catchments, can be used by planners to identify areas that, if protected, could provide the maximum benefits to coastal-marine ecosystems and species by preventing sedimentation in marine areas of high conservation priority [22, 33]. Likewise, further adjusting the management objectives (and associated river-plume exposure model) to reflect potential responses of marine ecosystems (in this case to increased sedimentation; e.g., [2]) could significantly improve the formulation of objectives and the utility of planning outputs to guide management decisions.

### Is it possible to protect areas in catchments that contribute to both local (terrestrial) and downstream (marine) management objectives?

Our findings highlight for the terrestrial realm what other studies have found for marine conservation priorities when land-based impacts were considered [41, 92]. We found important differences in the spatial distribution of land values derived from terrestrial and marine objectives, just as Tallis et al. [92] found differences in marine priorities when land-based threats were ignored or avoided. The minimal congruence between the priorities for conserving land vertebrates and protecting native vegetation to maintain water quality illustrates competing objectives (hence trade-offs) in integrated catchment management. Similarly, Klein et al. [33] found potential trade-offs between protecting native forests to achieve terrestrial conservation objectives and to provide downstream benefits to coral reefs.

Not surprisingly, similarity between the priorities for improving water quality through best-practice management and protection against potential erosion was also minimal. In this case, spatial differences were indicative of the targeted features and the types of actions to be implemented to achieve objectives. The first scenario involved improving management in sub-catchments dominated by anthropic land uses (e.g., through the implementation of agricultural best-practice management; see [78, 79]) and the other involved the protection of native vegetation to avoid clearing and hence erosion [21, 33]. Despite the marked differences between our scenarios (and associated trade-offs between management objectives), there are spatial options to manage areas to achieve local and downstream objectives simultaneously (i.e., management co-benefits: [25]). This is a promising result, and indicates that integrated land-sea planning can help to integrate and accommodate multiple objectives with benefits for both terrestrial and marine biodiversity, while reducing the overall cost of interventions [31].

### Study limitations and further research

While our study does not yet reach full integration of land and sea conservation–which would involve simultaneous planning to achieve objectives for land and adjacent marine waters and their interactions [31]–it represents an important advance in land-sea planning and proposes a method that can inform decisions about catchment management at the relevant spatial scales using readily available data. Our study had three key limitations. First, we provided only a cursory link between end-of-river loads of pollutants and the marine environment [82]. The model of exposure to river plumes proposed by Devlin et al. [93], including recent improvements [94–96], is a good alternative to be explored for the Gulf of California. Unfortunately, implementing the suggested exposure model in the Gulf will require additional adaptations. For instance, it would necessitate differentiating chlorophyll-rich river-plume waters from periodic upwelling of nutrient-rich water resulting in colourful phytoplankton blooms unrelated to catchments [54] and developing a new set of spectral signatures adapted to the region.

A second important limitation of our study is that we used pre-existing marine conservation priorities instead of targeting marine and terrestrial conservation areas simultaneously. However, this approach was necessary to accommodate previously established marine priorities resulting from an extensive consultative marine planning process. The marine spatial plan for the Gulf of California currently guides actions for conservation and management of coastal-marine areas in the region [52]. Fully integrated land-sea planning would ideally determine marine and terrestrial priorities simultaneously, so that maximum benefits of conservation and management actions can be achieved for land and sea (and preferably for freshwater ecosystems too), and costs of interventions can be minimised across realms. A next step to simultaneously minimise land-based threats and prioritise marine interventions may require adapting conservation planning tools to explicitly incorporate connectivity between land and marine planning units (some options to adapt conservation planning tools to account for connectivity are provided by: [43, 97, 98]).

Finally, we recognise inherent limitations following the integration of multiple (and sequential) models into prioritisation analyses. Quantifying and accounting for uncertainty in model outputs (which can be compounded across models) is needed to communicate the reliability of results to decision-makers, and could avoid suboptimal allocation of management actions [99]. Therefore, our suggested approach could be improved through the use of sensitivity analyses [100], scenario planning [101], and simulation modelling [102], which are useful tools to explore the consequences of using different parameters, models or management alternatives, and can facilitate integrated modelling [22, 103], particularly when data are missing or uncertain. Furthermore, undertaking cost-benefit analyses across management scenarios can further inform and support decisions [104, 105]; particularly, managers need guidance in weighing local and downstream environmental benefits against the socioeconomic costs (and benefits) to agriculture and other industries.

## Conclusion

The need to integrate land and marine conservation planning has been pointed out by several studies [23, 92, 97], yet to date very little emphasis has been placed on examining terrestrial management for achieving marine conservation objectives [24, 31]. Our study further advanced current approaches to land-sea planning [21, 22, 33] through developing a method that can guide planners (at a resolution amenable to management actions) to prioritise areas for management or protection to achieve terrestrial and marine conservation objectives. Our study proposes a method that integrates modelling of catchments, land-use change, and river-plumes with conservation planning software to prioritise management actions. While the modelling required to achieve this kind of land-sea conservation integration is complex and time-consuming [19, 106, 107], we show that it is possible and feasible, but also necessary to better guide integrated land-sea planning.

## Supporting Information

S1 Fig Integration of models and analysis for land-sea planning (PDF)

S1 Text Pollutant event mean concentrations used in catchment model (PDF)
