# Supplementary material for: Advancing Land-Sea Conservation Planning: Integrating Modelling of Catchments, Land-Use Change, and River Plumes to Prioritise Catchment Management and Protection
Source: PLoS One. 2015 Dec 29;10(12):e0145574. doi: 10.1371/journal.pone.0145574 (PMC4695094; doi:10.1371/journal.pone.0145574)
Supplement: S1 Fig — (PDF) [file pone.0145574.s001.pdf]

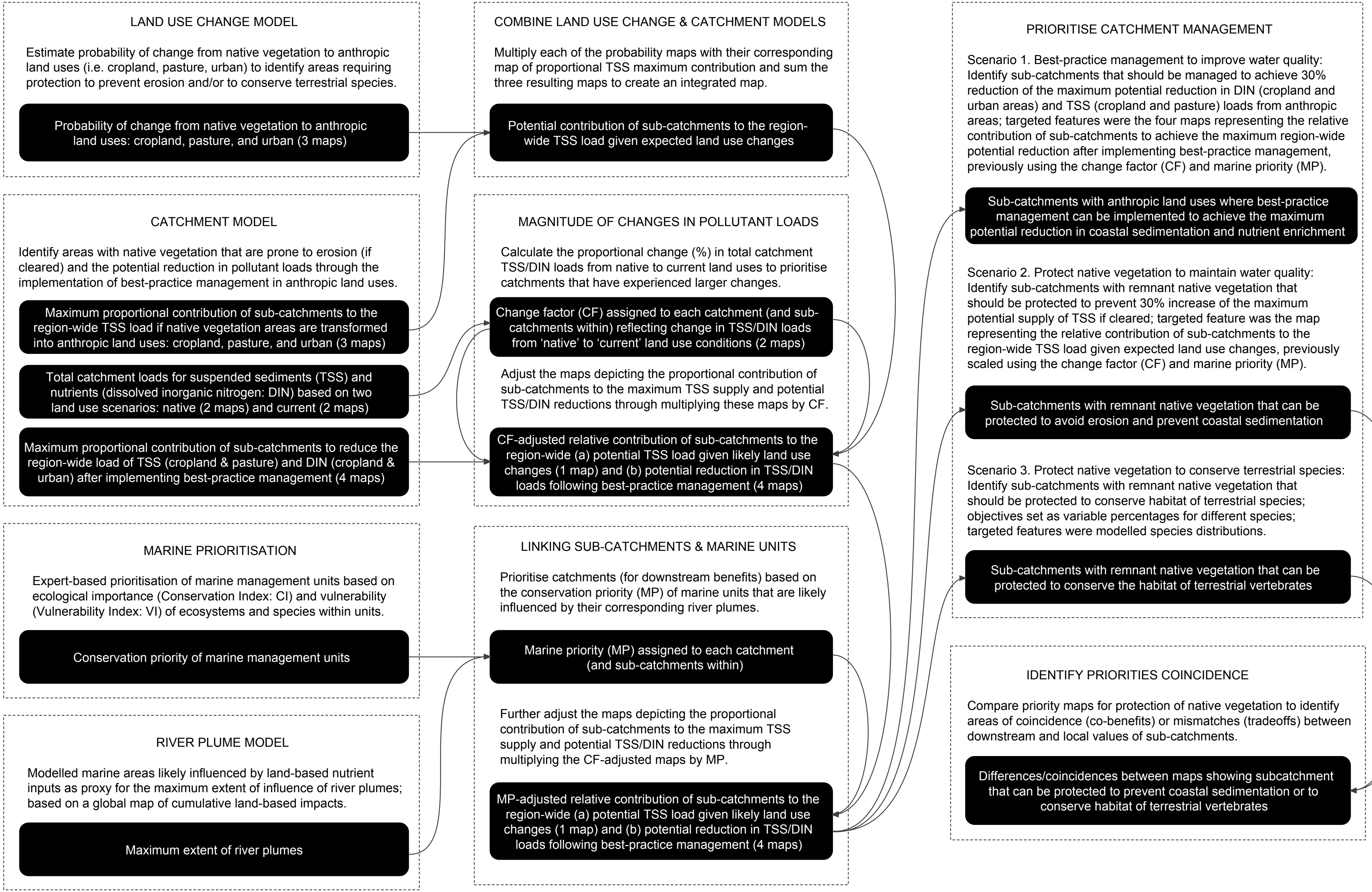
LAND USE CHANGE MODEL
Estimate probability of change from native vegetation to anthropic land uses (i.e. cropland, pasture, urban) to identify areas requiring protection to prevent erosion and/or to conserve terrestrial species.
Probability of change from native vegetation to anthropic land uses: cropland, pasture, and urban (3 maps)
CATCHMENT MODEL
Identify areas with native vegetation that are prone to erosion (if cleared) and the potential reduction in pollutant loads through the implementation of best-practice management in anthropic land uses.
Maximum proportional contribution of sub-catchments to the region-wide TSS load if native vegetation areas are transformed into anthropic land uses: cropland, pasture, and urban (3 maps)
Total catchment loads for suspended sediments (TSS) and nutrients (dissolved inorganic nitrogen: DIN) based on two land use scenarios: native (2 maps) and current (2 maps)
Maximum proportional contribution of sub-catchments to reduce the region-wide load of TSS (cropland & pasture) and DIN (cropland & urban) after implementing best-practice management (4 maps)
MARINE PRIORITISATION
Expert-based prioritisation of marine management units based on ecological importance (Conservation Index: CI) and vulnerability (Vulnerability Index: VI) of ecosystems and species within units.
Conservation priority of marine management units
RIVER PLUME MODEL
Modelled marine areas likely influenced by land-based nutrient inputs as proxy for the maximum extent of influence of river plumes; based on a global map of cumulative land-based impacts.
Maximum extent of river plumes
COMBINE LAND USE CHANGE & CATCHMENT MODELS
Multiply each of the probability maps with their corresponding map of proportional TSS maximum contribution and sum the three resulting maps to create an integrated map.
Potential contribution of sub-catchments to the region-wide TSS load given expected land use changes
MAGNITUDE OF CHANGES IN POLLUTANT LOADS
Calculate the proportional change (%) in total catchment TSS/DIN loads from native to current land uses to prioritise catchments that have experienced larger changes.
Change factor (CF) assigned to each catchment (and sub-catchments within) reflecting change in TSS/DIN loads from 'native' to 'current' land use conditions (2 maps)
Adjust the maps depicting the proportional contribution of sub-catchments to the maximum TSS supply and potential TSS/DIN reductions through multiplying these maps by CF.
CF-adjusted relative contribution of sub-catchments to the region-wide (a) potential TSS load given likely land use changes (1 map) and (b) potential reduction in TSS/DIN loads following best-practice management (4 maps)
LINKING SUB-CATCHMENTS & MARINE UNITS
Prioritise catchments (for downstream benefits) based on the conservation priority (MP) of marine units that are likely influenced by their corresponding river plumes.
Marine priority (MP) assigned to each catchment (and sub-catchments within)
Further adjust the maps depicting the proportional contribution of sub-catchments to the maximum TSS supply and potential TSS/DIN reductions through multiplying the CF-adjusted maps by MP.
MP-adjusted relative contribution of sub-catchments to the region-wide (a) potential TSS load given likely land use changes (1 map) and (b) potential reduction in TSS/DIN loads following best-practice management (4 maps)
PRIORITISE CATCHMENT MANAGEMENT
Scenario 1. Best-practice management to improve water quality: Identify sub-catchments that should be managed to achieve 30% reduction of the maximum potential reduction in DIN (cropland and urban areas) and TSS (cropland and pasture) loads from anthropic areas; targeted features were the four maps representing the relative contribution of sub-catchments to achieve the maximum region-wide potential reduction after implementing best-practice management, previously using the change factor (CF) and marine priority (MP).
Sub-catchments with anthropic land uses where best-practice management can be implemented to achieve the maximum potential reduction in coastal sedimentation and nutrient enrichment
Scenario 2. Protect native vegetation to maintain water quality: Identify sub-catchments with remnant native vegetation that should be protected to prevent 30% increase of the maximum potential supply of TSS if cleared; targeted feature was the map representing the relative contribution of sub-catchments to the region-wide TSS load given expected land use changes, previously scaled using the change factor (CF) and marine priority (MP).
Sub-catchments with remnant native vegetation that can be protected to avoid erosion and prevent coastal sedimentation
Scenario 3. Protect native vegetation to conserve terrestrial species: Identify sub-catchments with remnant native vegetation that should be protected to conserve habitat of terrestrial species; objectives set as variable percentages for different species; targeted features were modelled species distributions.
Sub-catchments with remnant native vegetation that can be protected to conserve the habitat of terrestrial vertebrates
IDENTIFY PRIORITIES COINCIDENCE
Compare priority maps for protection of native vegetation to identify areas of coincidence (co-benefits) or mismatches (tradeoffs) between downstream and local values of sub-catchments.
Differences/coincidences between maps showing subcatchment that can be protected to prevent coastal sedimentation or to conserve habitat of terrestrial vertebrates
