# Supplementary material for: Advancing Land-Sea Conservation Planning: Integrating Modelling of Catchments, Land-Use Change, and River Plumes to Prioritise Catchment Management and Protection
Source: PLoS One. 2015 Dec 29;10(12):e0145574. doi: 10.1371/journal.pone.0145574 (PMC4695094; doi:10.1371/journal.pone.0145574)
Supplement: S1 Text — (PDF) [file pone.0145574.s002.pdf]

S2. Pollutant event mean concentrations (EMC) used in catchment models

Studies varied significantly in the methods used (although many didn't report methods at all), the constituents reported (most studies included N, but both TN and TP were commonly reported constituents, and only a few were comprehensive in reporting different fractions, including Din, DIP, DON and DOP). When not directly reported, we calculated TN or TP, as well as DIN and DIP, based on reported fractions. In many cases it was not possible to know if reported values were true EMC or other types of concentrations (see Bartley et al. 2011 for definitions and methods for estimating EMC).

Following, we present a summary of the values used for modelling in SedNet (see **Table** below), as well as the rational underlying our selection/calculation of values. We incorporated the concentrations used to construct best-practice management scenarios. These values were set to represent maximum potential reductions in nutrient runoff based on reported reductions from literature. For cropland classes, the value represents a reduction of 40% in EMC concentrations following fertilizer best practices. Since potential reductions are higher for areas were fertilizer use is more intense, we gradually reduced concentrations to match the cropland class (very high: 40%; high: 30%; moderate: 20%; Low: 10%; Very low: 5% reduction). We assumed that agricultural practices will have minor effects on DON and DOP concentration and thus EMC values for these fractions were not modified. For urban areas it corresponds to a maximum 20% reduction. For pasture, the considered management practices possibly have minor impact on nutrient concentrations, but can help to reduce erosion; same as for agriculture (these modifications were incorporated by modifying the [1]cover factor "C' used in RUSLE).

Rationale for selection/calculation of EMC values

Urban: We selected high density residential/dense urban, because most areas classified as urban areas in the land-cover/vegetation maps correspond to these classes and industrial/commercial, not to rural or sparse urban areas. When data was not available for the nutrient fraction/constituent we used aggregated values as noted. Since this class is the one with higher EMC values, we consider this to be an adequate/conservative approach. However, a nation-wide study in the USA shows that differences between classes are minimal and not significant. Also, since many urban areas have "deficient" sewage systems, we would expect that using a class with relatively higher EMC values would partially compensate for the lack of point source data.

Cropland (5 classes): There are significant differences in EMC values from different crops, mostly as a result from differences in fertilizer use. Since we don't have a map that identifies the crop types within the cropland classes for each period (1973, 1993 and 2000), we overlaid a map that classifies municipalities based on the average use of agrochemicals (fertilizers and pesticides) in five levels, from very low to very high (SEMARNAT-INE 2000) with the cropland areas from each land cover map. We used this classification as a proxy for fertilizer use and to subdivide the class corresponding to cropland in five subclasses. Following, we calculated the 50th, 60th, 70th, 80th, and 90th percentiles of all the available EMC values to represent the classes with different levels of fertilizer/agrochemicals use. When calculated values were the same for different cropland classes or when values were lower than those for native vegetation classes, these were adjusted accordingly.

Grassland (native grasslands): We used the median value of vegetation identified as native grasslands of the full dataset, except in cases when data was not available for a particular constituent or when values between native and modified/human-induced grasslands were minimal or inverted (i.e., higher for native vegetation conditions). For both the Australian and North American datasets, this class sometimes also contained cattle grazed areas. For the USA it includes grassland/herbaceous, and for Australia it includes savannah woodland/dryland sheep/cattle grazing in native vegetation conditions, all of which have a dominant grassland understory.

[1] Cover factor was also used to modify the weighting surface when projecting potential erosion from new urban areas under development. Since erosion from existing urban areas is relatively small, the cover factor assigned for past and current scenarios is relatively small; however, erosion in areas under development can be significant, so the highest C factor (i.e., bare land = 0.700) was assigned to these areas. For best management practices, we assumed a maximum reduction of 30% from cropland and 20% from pasture, thus used 0.1827 and 0.184, respectively.

Pasture (modified/induced grasslands): We used the median value of vegetation identified as modified grasslands of the full dataset, except in cases when data was not available for a particular constituent or when values between native and modified/human-induced grasslands were minimal or inverted (i.e., higher for native vegetation conditions). Classes in the USA include pasture/hay, and for Australia, it also includes dairy cow pasture grazing and intensive grazing areas.

Forests: Due to difficulty in matching/cross-walking our land cover/vegetation classes to diverse forest classes reported in EMC studies and to high similarity and inconsistencies in the variation in EMC values between different types of forests for different constituents we decided to aggregate EMC values for all types of forests, including dry scrub/shrub and xeric vegetation. We thus assign gradually lower values, starting from temperate forests, down to xeric/desert environments, based on calculated percentiles (i.e., 90th to 25th). Higher values correspond to woodlands (temperate coniferous and mixed forest), followed by rainforests (humid tropical forests), dry forests (thorn forests and bushland), and scrub/shrub (xeric desert and semi-desert vegetation). We assigned the values of "rain forest" to "palm forest" due to its proximity and due to the minimal area that it occupies within the study region. Until we have more information specific for Mexico, this could be considered a working hypothesis to be tested using local experimental/observational data.

Although we would expect that erosion in areas with poor vegetation cover (e.g, scrub-shrub, xeric vegetation), would be important and therefore particulate nutrient concentrations would be higher, studies indicate that arid and xeric environments tend to export significantly less nutrients than temperate and rain forests (Perakis and Hedin 2002; Smith et al., 2003). Smith et al. (2003) estimated natural background concentrations for vegetation types that correspond fairly well with vegetation classes in our study region and showed that those corresponding to arid environments are significantly lower than those corresponding to temperate forests.

Other land cover/vegetation classes: Information for EMC values for other land cover/vegetation classes (e.g., wetlands, coastal dunes) was very scarce and values very contrasting. Considering they represent a minor portion of the region and that expected nutrient runoff from these areas is very low, either because runoff is very low or because they act like reservoirs for nutrients from upstream sources, we set values to zero for these classes. In contrast, values for bare/exposed land (i.e., cleared areas, mostly for urban or agricultural areas) were set high based on available studies, mostly only form North American studies.

References

Perakis, S.S., Hedin, L.O., 2002. Nitrogen loss from unpolluted South American forests mainly via dissolved organic compounds. Nature 415, 416-419.

SEMARNAT-INE, 2000. Índice de uso de agroquímicos por municipio, 1991. Secretaría del Medio Ambiente y Recursos Naturales, Instituto Nacional de Ecología, Dirección General de Ordenamiento Ecológico e Impacto Ambiental, Mexico, D.F.

Smith, R.A., Alexander, R.B., Schwarz, G.E., 2003. Natural background concentrations of nutrients in streams and rivers of the conterminous United States. Environmental Science & Technology 37, 3039-3047.

Nutrient runoff concentrations (mg/L) used for catchment models in the Gulf of California

Grey numbers are the maximum and minimum values contained in the reviewed studies.

Red numbers indicate the criteria to assign values were adjusted, e.g., when percentile values were the same for different classes.

Blue numbers indicate concentrations used to construct best-practice management scenarios.

Class	TN	DIN	DON	TP	DIP/FRP	DOP
Urban	[2]All (median high density residential)<br>**2.020 / 1.616**<br>(1.200-2.220)	All (median high density residential)<br>**0.794 / 0.635**<br>(0.200-1.650)	.[3]AUS (median aggregated)<br>**0.300 / 0.240**<br>(0.270-0.300)	All (median high density residential)<br>**0.470 / 0.376**<br>(0.200-0.570)	All (median high density residential)<br>**0.312 / 0.2496**<br>(0.143-0.480)	AUS (median aggregated)<br>**0.014 / 0.011**<br>(0.010-0.020)
Cropland<br>(5 classes)	All (50th to 90th percentiles)<br>Very high…**6.438/3.863**<br>High……...**5.409/3.786**<br>Moderate....**3.806/3.045**<br>Low……....**2.680/2.412**<br>Very low….**2.340/2.223**<br>(1.100-41.497)	All (50th to 90th percentiles)<br>Very high...**1.500/0.900**<br>High……..**1.047/0.733**<br>Moderate...**0.850/0.680**<br>Low……..**0.750/0.675**<br>Very low...**0.700/0.665**<br>(0.369-2.000)	AUS (50th to 90th percentiles)<br>Very high...**0.300**<br>High……..**0.275**<br>Moderate...**0.250**<br>Low……...**0.200**<br>Very low….**0.200**<br>(0.200-0.502)	All (50th to 90th percentiles)<br>Very high....**1.348/0.809**<br>High……...**1.134/0.794**<br>Moderate…**0.420/0.336**<br>Low………**0.371/0.334**<br>Very low….**0.340/0.323**<br>(0.140-2.294)	All (*ad hoc* values)<br>Very high....**0.090/0.060**<br>High……..**0.080/0.056**<br>Moderate…**0.070/0.055**<br>Low………**0.060/0.054**<br>Very low….**0.050/0.048**<br>(0.010-0.100)	All (50th to 90th percentiles)<br>Very high....**0.025**<br>High……...**0.020**<br>Moderate…**0.020**<br>Low………**0.019**<br>Very low….**0.014**<br>(0.002-0.027)
Pasture	All (modified)<br>**2.235**<br>(1.100-2.480)	All (modified)<br>**0.399**<br>(0.188-0.610)	AUS – all classes (90th)<br>**0.327**<br>(0.118-0.360)	All (modified)<br>**0.355**<br>(0.120-0.480)	All (both)<br>**0.033**<br>(0.013-0.050)	AUS - 1 value (modified)<br>**0.017**
Grassland	All (native)<br>**1.086**<br>(0.120-2.100)	All (native)<br>**0.190**<br>(0.160-0.200)	AUS – all classes (50th)<br>**0.225**<br>(0.118-0.360)	All (native)<br>**0.277**<br>(0.010-0.350)	All (.[4]both)<br>**0.033**<br>(0.013-0.050)	AUS (native)<br>**0.011**<br>(0.010-0.012)
Woodland	All (all classes including scrub/shrub: 90th percentile)<br>**0.700**<br>(0.050-0.830)	All (all classes including scrub/shrub: 90th percentile)<br>**0.170**<br>(0.040-0.180)	AUS (all classes including scrub/shrub: 90th percentile)<br>**0.227**<br>(0.072-0.257)	All (all classes including scrub/shrub: 90th percentile)<br>**0.052**<br>(0.006-0.070)	All (all classes including scrub/shrub: 90th percentile)<br>**0.037**<br>(0.004-0.050)	AUS (all classes including scrub/shrub: 90th percentile)<br>**0.013**<br>(0.005-0.016)
Rainforest	All (all classes including scrub/shrub: 75th percentile)<br>**0.669**<br>(0.050-0.830)	All (all classes including scrub/shrub: 75th percentile)<br>**0.134**<br>(0.040-0.180)	AUS (all classes including scrub/shrub: 75th percentile)<br>**0.208**<br>(0.072-0.257)	All (all classes including scrub/shrub: 75th percentile)<br>**0.043**<br>(0.006-0.070)	All (all classes including scrub/shrub: 75th percentile)<br>**0.028**<br>(0.004-0.050)	AUS (all classes including scrub/shrub: 75th percentile)<br>**0.010**<br>(0.005-0.016)
Dry forest/ Palm forest	All (all classes including scrub/shrub: 50th percentile)<br>**0.501**<br>(0.050-0.830)	All (all classes including scrub/shrub: 50th percentile)<br>**0.100**<br>(0.040-0.180)	AUS (all classes including scrub/shrub: 90th percentile)<br>**0.150**<br>(0.072-0.257)	All (all classes including scrub/shrub: 50th percentile)<br>**0.020**<br>(0.006-0.070)	All (all classes including scrub/shrub: 50th percentile)<br>**0.010**<br>(0.004-0.050)	AUS (all classes including scrub/shrub: 25th percentile)<br>**0.009**<br>(0.005-0.016)
Scrub-Shrub/ Halophytic	All (all classes including scrub/shrub: 25th percentile)<br>**0.222**<br>(0.050-0.830)	All (all classes including scrub/shrub: 90th percentile)<br>**0.051**<br>(0.040-0.180)	AUS (all classes including scrub/shrub: 90th percentile)<br>**0.110**<br>(0.072-0.257)	All (all classes including scrub/shrub: 25th percentile)<br>**0.011**<br>(0.006-0.070)	All (all classes including scrub/shrub: 75th percentile)<br>**0.007**<br>(0.004-0.050)	AUS (all classes including scrub/shrub: 10th percentile)<br>**0.007**<br>(0.005-0.016)
Wetland / Water	0	0	0	0	0	0
Dunes	0	0	0	0	0	0
Bare land	NA<br>**1.500**<br>(0.970-7.550)	NA<br>**0.665**<br>(0.540-0.790)	0	NA<br>**0.200**<br>(0.100-0.590)	NA<br>**0.030**<br>(Only value)	0

Reviewed studies for EMC values

Adamus & Bergman (1995); Mean runoff concentrations; Reference: Adamus, C. L. and M. J. Bergman (1995). "Estimating Nonpoint Source Pollution Loads with a GIS Screening Model." Journal of the American Water Resources Association 31(4): 647-655.

Baird & Jennings (1996); EMC median values; Reference: Baird, C. and M. Jennings (1996). Characterization of nonpoint sources and loadings to Corpus Christi Bay National Estuary Program Study Area. Austin, Texas, Texas Natural Resource Conservation Commission.

Bartley & Spears (2010; 2011); EMC median values (>90% indicated land use); Reference: Bartley R, Speirs W, Ellis T, Waters DK. 2011. A review of sediment and nutrient concentration data from Australia for use in catchment water quality models. Marine Pollution Bulletin .

---

[2] All = Values used were taken from full dataset, which included mostly studies from North America and Australia.

[3] AUS = When values were not available from North American studies or were not reliable or corrected (e.g., for forested areas where atmospheric deposition is expected to be high and therefore values were much higher than those reported for pristine systems), values from Australia and Americas (not USA) were used.

[4] Values for native grassland and pasture were very similar (higher for grassland), so we used the same value for both.

Bartley & Spears (2010; 2011); EMC median values; Reference: Bartley R, Speirs W, Ellis T, Waters DK. 2011. A review of sediment and nutrient concentration data from Australia for use in catchment water quality models. Marine Pollution Bulletin .

Brodie & Mitchell (2005): We calculated average from ranges): Reference: Brodie J, Mitchell A. 2005. Nutrients in Australian tropical rivers: changes with agricultural development and implications for receiving environments. Marine and Freshwater Research 56: 279-302

Brodie et al. (2003); EMC 80th percentile values; Reference: Brodie J, McKergow LA, Prosser IP, Furnas M, Hughes AR, Hunter H. 2003. Sources of Sediment and Nutrient Exports to the Great Barrier Reef World Heritage Area ACTFR Report No. 03/11, Australian Centre for Tropical Freshwater Research, James Cook University - CSIRO Land and Water, Townsville, QLD.

Cogle et al. (2006); EMC mean values; Reference: Cogle AL, Carroll C, Sherman BS, eds. 2006. The use of SedNet and ANNEX models to guide GBR catchment sediment and nutrient target setting. Queensland, Australia: Department of Natural Resources, Mines and Water.

Ellis et al. (2005); EMC median values (full dataset); Reference: Ellis T, Bartley R, Rahman J, Weber T, Henderson A, et al. 2005. Application of two water quality models as a Decision Support system (DSS) within the Douglas Shire, CSIRO Land and Water, Canberra, ACT.

EPA (1983); EMC 90th percentile values; Reference: U.S. Environmental Protection Agency (EPA). 1983. Results of the Nationwide Urban Runoff Program, Volume I - Final Report. Water Planning Division, Washington, D.C.

EPA (1983); EMC median values; Reference: U.S. Environmental Protection Agency (EPA). 1983. Results of the Nationwide Urban Runoff Program, Volume I - Final Report. Water Planning Division, Washington, D.C.

Furnas (2003): EMC median concentrations; Reference: Brodie J, Mitchell A. 2005. Nutrients in Australian tropical rivers: changes with agricultural development and implications for receiving environments. Marine and Freshwater Research 56: 279-302.

Hollinger & Cornish (2002); EMC median values; Reference: Drewry JJ, Newham LTH, Greene RSB, Jakeman AJ, Croke BFW. 2006. A review of nitrogen and phosphorus export to waterways: context for catchment modelling. Marine and Freshwater Research 57: 757-74.

Lewis et al. (2002): References: Lewis WM, Melack JM, McDowell WH, McClain M, Richey JE. 1999. Nitrogen yields from undisturbed watersheds in the Americas. Biogeochemistry 46: 149-62; and Lewis WM. 2002. Yield of nitrogen from minimally disturbed watersheds of the United States. Biogeochemistry 57: 375-85.

Line et al. (2002); EMC mean values; Reference: Line DE, White NM, Osmond DL, Jennings GD, Mojonnier CB. 2002. Pollutant Export from Various Land Uses in the Upper Neuse River Basin. Water Environment Research 74: 100-08.

Line et al. (2002); EMC median values; Reference: Line DE, White NM, Osmond DL, Jennings GD, Mojonnier CB. 2002. Pollutant Export from Various Land Uses in the Upper Neuse River Basin. Water Environment Research 74: 100-08.

L-THIA (default values): EMC values; Reference: Nejadhashemi AP, Mankin KR. 2007. Comparison of Four Water Quality Models (STEPL, PLOAD, L-THIA, and AVSWAT-X) in Simulating Sediment and Nutrient Dynamics in a Watershed. Presented at ASABE Annual International Meeting, Minneapolis, Minnesota

Meixler & Bain (2010): EMC values taken from other studies and adjusted; Reference: Meixler M, Bain M. 2010. A Water Quality Model for Regional Stream Assessment and Conservation Strategy Development. Environmental Management 45: 868-80.

Moller et al. (2005): concentrations; Reference: Moller A, Kaiser K, Guggenberger G. 2005. Dissolved organic carbon and nitrogen in precipitation, throughfall, soil solution, and stream water of the tropical highlands in northern Thailand. Journal of Plant Nutrition and Soil Science-Zeitschrift Fur Pflanzenernahrung Und Bodenkunde 168: 649-59.

Nelson et al. (1006); EMC median values; Reference: Drewry JJ, Newham LTH, Greene RSB, Jakeman AJ, Croke BFW. 2006. A review of nitrogen and phosphorus export to waterways: context for catchment modelling. Marine and Freshwater Research 57: 757-74.

Newell et al. (1992); EMC median values; Reference: Newell, C.J., H.S. Rifai, and P.B. Bedient. 1992. Characterization of Non-Point Sources and Loadings to Galveston Bay. Publication GBNEP-15, Galveston Bay National Estuary Program, Webster, Texas.

N-SPECT (default values): EMC values; Reference: NOAA. 2008. Nonpoint-Source Pollution and Erosion Comparison Tool (N-SPECT): Technical Guide, Ver. 1.5 (NOAA/CSC/RPT 08-5), National Oceanic and Atmospheric Administration (NOAA), Coastal Services Center, Charleston, SC

Omernick (1977) - Central: EMC values; Reference: Omernik JM. 1977. Nonpoint source-stream nutrient level relationships: a nationwide study, U.S. EPA Report No. EPA-600/3-77-105, U.S. Environmental Protection Agency, Corvallis, Oregon

Omernick (1977) - East: EMC values; Reference: Omernik JM. 1977. Nonpoint source-stream nutrient level relationships: a nationwide study, U.S. EPA Report No. EPA-600/3-77-105, U.S. Environmental Protection Agency, Corvallis, Oregon

Omernick (1977) - West: EMC values; Reference: Omernik JM. 1977. Nonpoint source-stream nutrient level relationships: a nationwide study, U.S. EPA Report No. EPA-600/3-77-105, U.S. Environmental Protection Agency, Corvallis, Oregon

Perakis & Hedin (2002): Perakis SS, Hedin LO. 2002. Nitrogen loss from unpolluted South American forests mainly via dissolved organic compounds. Nature 415: 416-19.

PLOAD (default values): EMC values; Reference: Nejadhashemi AP, Mankin KR. 2007. Comparison of Four Water Quality Models (STEPL, PLOAD, L-THIA, and AVSWAT-X) in Simulating Sediment and Nutrient Dynamics in a Watershed. Presented at ASABE Annual International Meeting, Minneapolis, Minnesota

Smith et al. (2003): Modelled natural background concentrations for nutrient ecoregions; Reference: Smith RA, Alexander RB, Schwarz GE. 2003. Natural background concentrations of nutrients in streams and rivers of the conterminous United States. Environmental Science & Technology 37: 3039-47.

Smullen et al. (1999): EMC mean values; Reference: Smullen JT, Shallcross AL, Cave KA. 1999. Updating the U.S. nationwide urban runoff quality database. Water Science Technology 39: 9-16.

Smullen et al. (1999): EMC median values; Reference: Smullen JT, Shallcross AL, Cave KA. 1999. Updating the U.S. nationwide urban runoff quality database. Water Science Technology 39: 9-16.

Strager (2000); EMC mean values; Reference: Strager MP, Fletcher JJ, Yuill CB, Strager JM. 2000. Not in my watershed! An interactive tool to evaluate land use changes on stream water quality. Presented at ESRI International Conference Proceedings, Redlands, CA.
